# Association of glutamine supplementation during the early suckling period with growth, weaning, and lipopolysaccharide induced stress in low birthweight piglets

**DOI:** 10.1093/jas/skaf296

**Published:** 2025-08-30

**Authors:** Miriama Sciascia, Johannes Buchallik-Schregel, Zeyang Li, Solvig Görs, Armin Tuchscherer, Torsten Viergutz, Andreas Höflich, Jürgen Zentek, Cornelia Christiane Metges

**Affiliations:** Research Institute for Farm Animal Biology (FBN), Dummerstorf, Mecklenburg-Vorpommern, Germany; Research Institute for Farm Animal Biology (FBN), Dummerstorf, Mecklenburg-Vorpommern, Germany; Research Institute for Farm Animal Biology (FBN), Dummerstorf, Mecklenburg-Vorpommern, Germany; Research Institute for Farm Animal Biology (FBN), Dummerstorf, Mecklenburg-Vorpommern, Germany; Research Institute for Farm Animal Biology (FBN), Dummerstorf, Mecklenburg-Vorpommern, Germany; Research Institute for Farm Animal Biology (FBN), Dummerstorf, Mecklenburg-Vorpommern, Germany; Research Institute for Farm Animal Biology (FBN), Dummerstorf, Mecklenburg-Vorpommern, Germany; Ligandis UG, Gülzow-Prüzen, Mecklenburg-Vorpommern, Germany; Department of Veterinary Medicine, Institute of Animal Nutrition, Freie Universität Berlin, Berlin, Germany; Research Institute for Farm Animal Biology (FBN), Dummerstorf, Mecklenburg-Vorpommern, Germany

**Keywords:** glutamine, innate immune system, lipopolysaccharide, low birthweight, pigs, weaning

## Abstract

Glutamine (**Gln**) supplementation has been shown to improve bodyweight (BW) in suckling low birthweight (**LBiW**) pigs. However, it is not known if Gln has lasting effects on growth and stress resilience beyond the supplementation period in LBiW suckling pigs. Therefore, we explored if post-Gln supplementation LBiW pigs show reduced stress during weaning and a lipopolysaccharide challenge. Twenty pairs of male German Landrace littermate piglets, originating from 14 L with 12 to 22 (live and stillborn) piglets, were selected at birth (0 d of age). One littermate was LBiW (mean 1.10 ± 0.02 kg; *n *= 20; below the lowest BiW quartile of the FBN pig facility) and the other normal (**NBiW**; mean 1.48 ± 0.02 kg; *n* = 20; within the middle 50th BiW quartile) birthweight. At 24 h postfarrowing, litter sizes were standardized to 12 piglets, and experimental LBiW and NBiW piglets assigned to Gln (1 g/kg BW/d) or alanine (**Ala**: isonitrogenous control to Gln; 1.22 g/kg BW/d) supplementation groups (LBiW-Gln, LBiW-Ala, NBiW-Gln, and NBiW-Ala). Piglets were orally supplemented 3 times daily (0700, 1200, and 1700), from 1 to 12 d of age, and remained with their birth litter until weaning at 28 d of age. At 55 d of age, pigs were challenged with lipopolysaccharide (i.p. 100 µg/kg BW) and euthanized at 61 d of age. The piglets were weighed, and their abdominal circumference, crown-rump length, body mass index and ponderal index were determined. Plasma metabolites, amino acids, TNF-α, and white blood cell counts were also measured. At birth, LBiW pigs were lighter (*P* = 0.002), had a shorter crown-rump length (*P* = 0.02), a smaller abdominal circumference (*P* = 0.02), a lower body mass index (*P* < 0.001), and plasma glucose (*P* = 0.07) but higher inositol (*P* = 0.001) levels, than their NBiW littermates. From pre- (27 d) to postweaning (32 d), the lymphocyte percentage decreased, and the segmented neutrophil percentage and the neutrophil to lymphocyte ratio increased in LBiW-Ala (*P* < 0.001), NBiW-Ala and NBiW-Gln (*P* < 0.05) pigs. Postlipopolysaccharide-challenge, TNF-α was lower at 1 h in Gln than Ala-supplemented pigs (*P* < 0.05). In conclusion, LBiW piglets had zootechnical and metabolic markers associated with impaired development at birth, and supplementation with Gln moderately improved immune markers of stress during weaning, and reduced the TNF-α peak in LBiW and NBiW pigs during a lipopolysaccharide challenge. However, no effect on LBiW piglet bodyweight was observed.

## Teaser

Supplementation of low birthweight piglets from 1 to 12 d of age with the amino acid glutamine appears to reduce immune markers of stress during the weaning period and plasma TNF-α levels during a lipopolysaccharide challenge. Therefore, low birthweight piglets supplemented with glutamine may have adapted earlier to their new postweaning housing environment and social order than normal birthweight piglets, whilst glutamine supplemented piglets, independent of birthweight group, may have a reduced inflammatory response after a lipopolysaccharide challenge.

## Introduction

Low birthweight (**LBiW**) is caused by impaired fetal growth and development during gestation that can carry over into the postnatal period and prevent a piglet from reaching its full genetic potential (Huting et al., 2021). Postnatal impairments include reduced organ functionality and age-corrected bodyweight, increased disease risk and mortality at vulnerable life stages such as pre- and early postweaning (D’Inca et al., 2010; Hales et al., 2013; Ferenc et al., 2018), which appear to be especially prevalent in male pigs (Van Ginneken et al., 2022). Therefore, targeted nutritional interventions such as pre- and probiotics, or fatty acids and amino acids (**AA**) have been used to reduce the impact of retarded in utero development on LBiW piglet postnatal growth, health, and development (Li et al., 2017).

One such targeted nutritional intervention is supplementation with Glutamine (**Gln**), the most abundant AA in sow’s milk; it has been reported to regulate the signaling pathways linked to growth, stress, and immunity (Li et al., 2017). Studies of Gln supplementation in pigs have shown that during the preweaning phase, it has a positive effect on growth (Haynes et al., 2009; Wu et al., 2011), and 2 companion studies conducted by our group confirm a moderate improvement in growth, during (Li et al., 2022) and post (Schulze Holthausen et al., 2022) supplementation. Additionally, improved milk intake and lipid metabolism were observed in LBiW pigs during the supplementation period (Li et al., 2022). Supplemental Gln and several Gln dipeptides (glycyl-Gln, alanyl-Gln) have also been reported to improve growth, intestinal morphology, enzymatic function, antioxidant status, and protein synthesis during periods of stress, such as weaning (Ji et al., 2019; Xiong et al., 2019) and immune stimulation with lipopolysaccharide (**LPS**) and bacterial infections (Jiang et al., 2009; Hsu et al., 2012; Xu et al., 2021). The effects of supplementation with Gln (Wang et al., 2008; Haynes et al., 2009; Wu et al., 2011) and other AA (Sun et al., 2015; Fan et al., 2019; Ji et al., 2023) have been investigated in most studies in comparison to an isonitrogenous control supplementation using Ala. Alanine is a nonessential amino acid that is relatively metabolically inert compared with other nonessential amino acids, which minimizes potential interference effects of control supplementation (Chambon-Savanovitch et al., 1999).

It has been reported that during the supplementation period additional dietary, Gln improves LBiW suckling piglet growth and development (Wu et al., 2011; Li et al., 2022), but there is a paucity of data on whether the effect is sustained after the supplementation period has ended. In a recent companion study performed by our group, improved growth ceased 9 d after the end of the supplementation (Schregel et al., 2024). We hypothesized that Gln supplementation would increase the bodyweight (BW) of LBiW piglets, reduce leukocyte and oxidized glutathione levels and alter plasma AA profiles during weaning and during an LPS challenge, and reduce plasma TNF-alpha concentrations during the LPS challenge. Therefore, this study investigated the influence of oral glutamine supplementation at 1 to 12 d of age in LBiW suckling piglets and determined the effects on growth, immune system, glutathione, and amino acid profiles during weaning and LPS challenge.

## Materials and Methods

### Ethics statement

The study was performed between January 2019 and April 2020, at the Research Institute for Farm Animal Biology’s (**FBN**) experimental pig facility. Ethical approval for all experimental procedures was obtained from the State Office for Agriculture, Food Safety and Fishery, Mecklenburg-Western Pomerania, Germany (permission No. 7221.3-1-026/16), and conducted according to the German Animal Welfare Act (Directive 2010/63/EU, protection of animals used for scientific purposes).

### Gilts and offspring housing conditions

German Landrace gilts (*n* = 14) were artificially inseminated at the onset of standing estrus and again 24 h later using semen sourced from three German Landrace boars (BVN Schweinebesamung Malchin GmbH, Malchin, Germany). Gestating gilts were managed in a dynamic group pen (15.9 m^2^), which held four animals at any one time (4.0 m² per gilt). The pen had a fully slatted concrete walking area, concrete lying bays paved with bedding (chopped straw, shavings, and spelt litter), and manually triggered feed troughs that were automatically filled into volumetric feeders via a feed chain, twice a day. Water was available ad libitum via basin and trough drinkers. At 28 d of age postinsemination, pregnancy was confirmed using an HD 6 portable veterinary ultrasound scanner (Caresono Technology Co, Shenzhen, China). Then ~10 d before gilts were due to farrow, they were moved into standard farrowing rooms, accommodating six animals per room and housed in individual farrowing pens. Farrowing room temperature was maintained at ~21 °C until the end of lactation (28 d of age), whilst the piglet nesting area had a heating plate (76 × 60 cm) and lamp, with computer-controlled temperature that started at 28 °C and was gradually reduced to 21 °C by 28 d of age (weaning). Artificial lighting in the farrowing room was provided daily from 0700 to 1515 h. Farrowing induction began 1 d prior to expected parturition (115 d of pregnancy), where gilts were injected *i.m.* with 2-mL Cloprostenol (Prostaglandin F2_alpha_, Veyx Pharma GmbH, Schwarzenborn, Germany), and on the day of parturition, 1-mL Carbetocin (Depotocin, Veyx Pharma GmbH). Litter characteristics were recorded immediately after birth and are reported in Supplementary Table 1. At 28 d of age, piglets were transferred to weaning pens and weaned in their respective supplementation groups (Gln or Ala, maximum 4 to 6 piglets per pen). The weaning pens (4.5 m^2^) had plastic floors with slats and underfloor heating, and piglets were fed via troughs. After weaning (33 d of age), piglets were transferred to individual pens (1.6 m²), which had concrete slatted flooring with integrated underfloor heating, and piglets were fed via troughs. In the weaning and individual pens, feed and water were available ad libitum, and heating was controlled by a climate-computer with a corresponding temperature curve.

### Gilt and pig diets

After insemination, gilts were fed a standard pregnancy diet (12.2 MJ of metabolizable energy [**ME**]/kg, 14.5% crude protein [**CP**]) until farrowing where they were fed a lactation diet (13.2 MJ ME/kg, 17.5% CP; both from Trede & v. Pein, Dammfleth, Germany), following German energy and nutrient recommendations for pregnant and lactating sows (GfE, 2006). Diets were fed twice a day (0730, 1400 h) according to the schedule in Supplementary Table 2.

Piglets were suckled by the respective dam until weaning (colostrum and milk composition; Supplementary Table 3) and had access to creep feed (Porcistart 14 MJ ME/kg, 18% CP; Trede & von Pein) from 14 to 27 d of age. Creep feed intake was not recorded as experimental piglets were kept in the same pen with their nonexperimental littermates, which confounded analysis of creep feed intake by the experimental animals. Postweaning (28 d of age) pigs had ad libitum access to the same standard commercial diets. From 28 to 32 d of age, pigs were provided with a starter diet (Turbostart, 15 MJ ME/kg, 19% CP; Trede & von Pein). At 33 d of age, pigs were introduced to a conventional pig starter diet (Porcistart 14 MJ ME/kg, 18% CP; Trede & von Pein). At 48 d of age, pigs were switched to a grower diet (Porcibig; 13.8 MJ ME/kg, 12.1% CP; Trede & von Pein).

### Piglets and experimental design

Initially, a total of 52 pairs (one LBiW and one normal birthweight [**NBiW**] piglet) of male littermates were selected during this study. Not all pairs could continue due to the following exclusion criteria used for this study, which were, 1) lost bodyweight (**BW**) for more than 2 consecutive days, 2) exhibited sickness symptoms (diarrhea, coughing) and required treatment, 3) lack of mobility, 4) died during the trial, 5) group was canceled due to insufficient numbers of animals, or 6) was an experimental litter mate to the excluded piglet (Supplementary Table 4). Thus, 20 pairs of male littermates, which were born to 14 gilts, spread across 9 farrowing dates, and sourced from litters with 12 to 22 (live and stillborn) piglets were subsequently selected to continue in the study (Supplementary Table 1). Male piglets were selected for this study, because they are reported to have a higher preweaning mortality rate than female littermates (Baxter et al., 2012; Chu et al., 2022) and to rule out possible sex-specific effects.

Immediately after birth (0 d of age), the umbilical cord was broken, the sex and birthweight (**BiW**) recorded in the farrowing pen, and the time until the first suckling was observed. Sixteen pairs of male littermates had a 0 h blood sample (before first suckling taken; see section “Blood sampling”). Not all piglets could be sampled due to the occurrence of multiple gilts farrowing at the same time.

In the 20 littermate pairs recorded, one littermate was LBiW (mean 1.10 ± 0.02 kg; below the lowest BiW quartile of the FBN pig facility) and the other NBiW (mean 1.48 ± 0.02 kg; within the middle 50th BiW quartile of the FBN pig facility) BiW. Each selected litter had 1-3 pairs of experimental piglets. At 24 h postfarrowing, litter sizes were standardized to 12 piglets, and experimental LBiW and NBiW piglets were assigned to Gln or an isonitrogenous alanine control (**Ala**) supplementation group (**Suppl**). Assignment to each AA group was performed so that the BW was not significantly different between piglets of the same BiW class. This resulted in four experimental groups (*n* = 10/group) with a mean BiW of LBiW-Gln (1.18 kg ± 0.19), LBiW-Ala (1.11 kg ± 0.19), NBiW-Gln (1.55 kg ± 0.19), and NBiW-Ala (1.46 kg ± 0.19).

**Figure 1.**
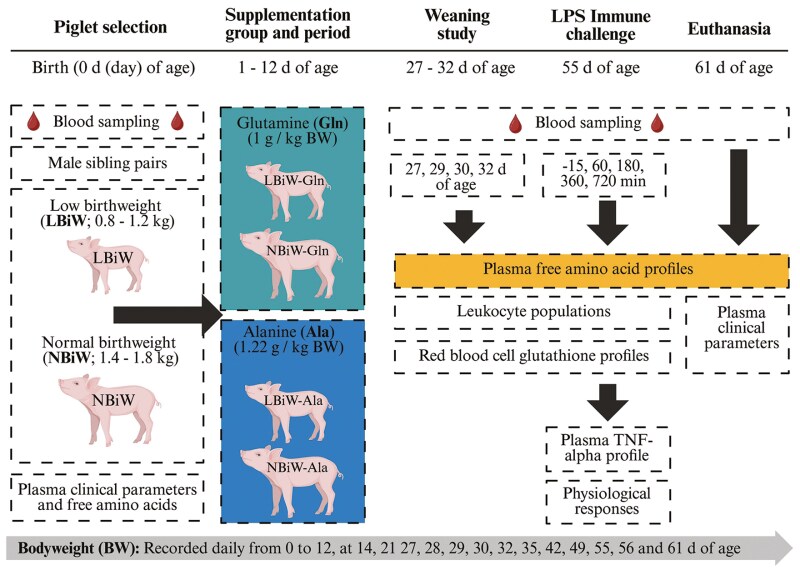
Experimental design showing postglutamine supplementation weaning and lipolysaccharide immune challenge studies in male piglets with low and normal birthweight supplemented with alanine (Ala) or glutamine (Gln) starting at 1 d of age^1^ (Created in BioRender). ^1^Birthweight of LBiW and NBiW piglets were 0.8–1.2 and 1.4–1.8 kg, respectively. At birth (0 d of age), LBiW and NBiW littermates were selected and the following day (1 d of age) randomly allocated to supplementation groups, resulting in 4 experimental groups (LBiW-Gln, LBiW-Ala, NBiW-Gln, NBiW-Ala). At 12 d of age, supplementation stopped. From 27 to 32 d of age, a weaning stress study (weaned at 28 d of age) was performed and at 55 d of age, a lipolysaccharide immune challenge (i.p, 100 µg/kg/bodyweight) was performed.

Supplemental AA was prepared the day prior to supplementation in 5-mL syringes, with each syringe containing one-third of the calculated daily dose. The daily dose was estimated using the BW (+200 g; estimated average preweaning daily BW gain for piglets in this study) and to ensure that the dose would be isonitrogenous (Gln; 1 g/kg BW/d or Ala; 1.22 g/kg BW/d). On the day of supplementation, Gln or Ala was diluted in 2 mL of water just prior to piglets being dosed. An additional 2 mL of water was used to rinse the syringes and ensure the entire that dose was given to the piglets. Piglets were orally supplemented three times daily (0700, 1200, and 1700) from 1 to 12 d of age (Li et al., 2022). The experimental overview can be found in Figure 1.

At 28 d of age, experimental pigs were weaned in their respective supplementation groups (Gln or Ala) and at 33 d of age transferred to individual pens for the remainder of the study. At 61 d of age, pigs were transported in pairs to the FBN slaughterhouse (journey time ~3 min), electro-stunned, and then euthanized via exsanguination.

BW was measured daily at ~0700, during supplementation period: 0 until 12 d of age, then at 14, 21 d of age, during the weaning study: 27, 28, 29, 30, and then 32, 42, 49 d of age, then on the day of the LPS study: 55 d of age, then 56 d of age and immediately prior to euthanasia (61 d of age). Abdominal circumference (**AC**), crown-rump length (**CRL**, the supine length of the piglet from the crown of its head to the base of its tail), and rectal temperature (**RT**) were measured, and body mass index (**BMI**, kg/m^2^) and ponderal index (**PI**, kg/m^3^) were calculated (Baxter et al., 2008), at birth (0 d of age), 5 d of age, weekly from 7 to 56 d of age and immediately prior to euthanasia. Time to suckle was calculated at birth as was defined as first teat contact where suckling occurs for more than 5 s (De Passille and Rushen, 1989).

### Colostrum composition and intake

Colostrum was collected within 2 h following birth of the first piglet and ~24 h postfarrowing. The 2 h samples were collected via hand stripping, whilst 24 h samples were collected after the sows were injected with 1-mL oxytocin (Longacton, carbetocine, IDT Biologika GmbH, Dessau–Roßlau). Both 2 and 24 h samples were collected from the same teat pairs, anterior (1st and 2nd pairs) and posterior (6th and 7th pairs), and equal volumes were pooled according to location sampled (anterior or posterior), and sampling time (4 or 24 h). Samples were stored at −20 °C for subsequent analysis of dry matter, crude fat, crude protein, lactose (Gors et al., 2009), and immunoglobulins (**Ig**) (IgA, IgM, and IgG) (Supplementary Table 3). The CV within and between assays for milk dry matter, crude fat, crude protein, lactose, and Ig was 0.7 and 1.5%, 2.6 and 2.3%, 5.1 and 1.4%, 5.1 and 6.4%, and 4.5 and 6.2%, respectively. Colostrum intake (**CI**) was calculated from birth (0 d of age) until 1 d of age using the BW gain over this period (Amdi et al., 2013).

### Blood sampling

At birth (0 h) and 4 h postbirth, 0.5 mL of blood was collected from a subpopulation of 16 littermate pairs (*n* = 32 piglets). At pre- (27 d of age) and postweaning (29, 30, and 32 d of age), at ~0800 (1.5 mL), on 55 d of age during the LPS test at −15 (baseline value), 60, 180, 360, and 720 min after LPS administration (1.5 mL), and immediately after euthanasia on 61 d of age (5 mL), blood samples were taken from the experimental piglets (*n* = 40). Birth, weaning, and LPS test blood sampling were conducted by placing all piglets in a supine position on a sampling table, and blood collected by anterior vena cava puncture using a needle and syringe, with the whole procedure lasting from 10 to 20 s. Slaughter samples were collected after the animal was euthanized. All blood samples were collected into K-EDTA tubes (Sarstedt, Germany). For the analysis of blood immune cells during pre- (27 d of age) and postweaning (29, 30, and 32 d of age) and the LPS challenge (55 d of age), an aliquot of whole blood was collected and kept at 4 °C, until analysis the next day. The remaining blood samples were centrifuged for 20 min at 1,576× g and 4 °C to obtain plasma and red blood cells (**RBC**). Plasma samples were stored at −80 °C for subsequent measurement of plasma clinical parameters, immunoglobulins (**Ig**), insulin-like growth factor 1 (**IGF-1**), insulin-like growth factors binding proteins 2, 3, 4, 5 (**IGFBP2-5**), free amino acids (**FAA**), and tumor necrosis factor-α (**TNF-α**). The RBCs were washed and stored as previously described (Rasch et al., 2020), with only modification being the RBCs were centrifuged at 1,600 g.

### LPS challenge and pig behavioral responses

At 55 d of age (beginning at 0600), all experimental pigs received an *i.p.* injection of 100 µg/kg BW LPS (*Escherichia coli* O111:B4; Sigma-Aldrich, Deisenhofen, Germany, Product No: L2630, Batch No: 028M4022V) dissolved in 3-mL sterile 0.9% NaCl. After LPS injection, sickness symptoms (Table 1) of 1) shivering, 2) impeded respiration, 3) vomiting, 4) diarrhea, and 5) circulation insufficiency and activity/inactivity (Tuchscherer et al., 2006; Wirthgen et al., 2018) were observed and recorded. Shivering, vomiting, and diarrhea were assessed by observing the entire animal, whilst circulation insufficiency was assessed by looking for a color change in the ears (cyanosis, skin with blueish appearance) and the skin (patchy white and pink areas). Impeded respiration was assessed by placing a hand at the bottom rib cage and feeling for a change in the breathing rhythm. Time points recorded were −15 min prior to, and every 15 min post-LPS injection until 360 min. A value of 1 was assigned to indicate the presence of a specific symptom, whereas a value 0 was assigned to denote its absence. Sickness severity was evaluated by summing the values of all sickness symptoms at each time point for each animal (minimum number = 0, maximum number = 5) and calculated as the mean value. Activity was characterized as active (1) or inactive (0). Then, the activity level was calculated as the percentage of the observations during the whole observation period of 6 h for each animal. Additionally, piglet RT and BW were recorded at −15, 60, 180, 360, 720, and 1,440 min relative to LPS injection. All pigs had ad libitum access to feed and water, prior to and during the LPS challenge.

**Table 1.** Parameters used to assess piglet sickness severity and activity/inactivity of male LBiW and NBiW piglets supplemented with alanine or glutamine, from 1 to 12 d of age, following an intraperitoneal injection of LPS (100 µg/kg bodyweight) at 55 d of age

Parameter	Behavior
Symptoms of sickness	(1) Shivering, (2) impeded respiration, (3) vomiting, (4) diarrhea, (5) circulatory insufficiency
Activity	Walking, drinking, feeding, drinking, or engagement with pig from the neighboring pen
Inactivity	Lying, sitting

### Differential and total white blood cell counts

Blood smears were prepared followed by air-drying. The object slide was then incubated for 2 min in May–Grünwald solution and washed with distilled water, followed by an incubation in Giemsa solution (1:20) for 30 min. After washing with distilled water, the slide was dried. To calculate the leukocyte distribution, 200 leukocytes were counted using light microscopy. The cell types were differentiated as lymphocytes (**LYM**), monocytes (**MONO**), and neutrophils with subdivision into banded (**Band**) and segmented (**Segs**) neutrophils (Niepage, 1974). Basophiles and eosinophils were counted; however, the numbers counted were low, so they could not be reliably analyzed, and thus removed from the study. As a marker for early acute inflammation and physiological stress, the neutrophil to lymphocyte count (**N/L**) ratio was also calculated (Wirthgen et al., 2018). Total white blood cells (**WBC**) were determined by conductometric counting (Coulter Counter; Coulter Electronics Inc., Hialeah, FL, USA) and cell viability was assessed by trypan blue exclusion, as recommended by the manufacturer (Sigma).

### Clinical parameters, free amino acids, and TNF-α in plasma

At birth (0 d), 4 h postbirth and 61 d of age, the concentrations of albumin (Kit No. A11A01664, Axon Lab AG, Reichenbach/Stuttgart, Germany), bilirubin (Kit No. A11A01639, Axon Lab AG), cholesterol (Kit No. 553–350, MTI Diagnostics, Idstein, Germany), glucose (Kit No. A11A01667 Axon Lab AG), urea (Kit No. LT-UR0010, Labor & Technik Eberhart Lehmann GmbH, Berlin, Germany), lactate (Kit No. A11A01721, Axon Lab AG), nonesterified fatty acids (**NEFA**: kit No. R1434–91795 and R2436–91995, WAKO Chemicals GmbH, Neuss, Germany), total protein (Kit No. 553–950, MTI Diagnostics) and triglycerides (**TG**: Kit No. A11A01640, Axon Lab AG), alanine aminotransferase (**ALT**: Kit No. A11A01627, Axon Lab AG), and aspartate aminotransferase (**AST**: Kit No. A11A01629, Axon Lab AG) were measured in all plasma samples using commercial kits and an automatic enzymatic analyzer (ABX Pentra 400, HORIBA Medical, Montpellier, France) (Das et al., 2016). The CV within and between assays for these measurements was <2.9% and <8%, respectively. The 61 d of age results are presented in Supplementary Table 5. Fructose and *myo*-inositol (henceforth referred to as inositol) were analyzed as previously reported (Metges et al., 2014), and the concentrations of IgA, IgG, and IgM were measured in duplicate with a porcine specific ELISA kit (Bethyl Laboratories Inc., Montgomery, TX) according to the manufacturer’s instructions, with a within and between CV of 2.3% and 4.4%, respectively. The detection range for each Ig was; IgA: 7.8 to 500, IgG: 15.6 to 1,000, and IgM: 15.6 to 1,000 (ng/mL). Insulin-like growth factor 1 was measured by ELISA (Mediagnost, Reutlingen, Germany: Product No: E20), and IGFBP2–5 were measured via Western ligand blot, as previously described (Wirthgen et al., 2016). For IGF-1, the CV within and between assays for these measurements were <1.6% and <3.7%, respectively. Free AA concentrations were determined, at birth (0 d of age), 4 h postbirth, pre- (27 d of age) and postweaning (29, 30 and 32 d of age), and during the LPS challenge (55 d of age), in plasma samples, 10-fold diluted with ultrapure water, based on Kromer et al. (2005), via HPLC with precolumn derivatization with ortho-phthalaldehyde (OPA)/3-mercaptopropionic acid (each 10 mg/mL in 0.4 M borate buffer [pH 10.2]) for primary and 9-fluorenylmethoxycarbonyl chloride (FMOC-Cl, 2.5 mg/mL in acetonitrile) for secondary amino acids, after using 3-mercaptopropionic acid as a reducing agent (0.5% in 0.5 M bicine buffer [pH 9.0]) and iodoacetic acid (50 mg/mL in bicine buffer) to block sulfhydryl groups. Separation was carried out on a 250 × 4.6 mm Gemini 5µm C18 110Å column with 4 × 3 mm precolumn (both Phenomenex, Aschaffenburg, Germany) at 40 °C and a flow of 0.8 mL/min. Derivatives were detected by fluorescence (OPA: excitation 340 nm, emission 450 nm; FMOC: excitation 266 nm, emission 305 nm). For quantification, an external calibration was performed using standard mixtures of amino acids (A9906, Sigma, Munich, Germany). The TNF-α concentrations were determined during the LPS challenge (55 d of age), with a porcine specific ELISA kit (R&D Systems Europe, Abington, UK: Product No: PTA00) according to the manufacturer’s instructions, with a within CV of 3.4 % and a between CV of 5.0 %.

### Erythrocyte GSH and GSSG concentrations

Red blood cells sampled during the weaning test (27 to 32 d of age) and the LPS test (55 d of age) were used to measure the concentrations of reduced (**GSH**) and oxidized (**GSSG**) glutathione as fluorescent ortho-phthaldialdehyde derivatives by HPLC according to Rasch et al. (2020) modified by the use of a 250 × 4.6 mm Gemini 5µm C18 110Å column with 4 × 3 mm precolumn on an Series 1260 infinity II system (Agilent) and another gradient for elution (eluent A: ACN/methanol/water = 45:45:10; eluent B: 40 mM phosphate buffer pH 7.45; 0 min—7% A, 4 min—14% A, 8 min—15% A, 15 min—16% A, and 20 min—100% A) to enable sufficient separation. Total GSH concentration was calculated as the sum of GSH and GSSG concentrations.

### Statistical analysis

Sample size (*n*) was calculated as a 2-level factor combination of 1) BiW (LBiW, NBiW) and 2) supplementation (Ala, Gln), using CADEMO for Windows ANOV-Version 4.03 (2000; BioMath GmbH, Rostock). Alpha or “the significance cut off” was set at 0.05, and beta or “failure to reject a false null hypothesis” was set at 0.20. The primary outcome measures used to determine *n* were BW gain and changes in blood stress markers in response to an LPS injection.

Model 1, used for the evaluation of colostrum composition, was an Analysis of Variance (ANOVA) using the MIXED procedure of SAS which contained the fixed effects of Teat Pair (1, 2 and 6, 7) and Time (2 and 24 h). The SLICE statement was used for performing partitioned analyses of the least-squares means for the teat pair × Time interaction. Model 2, used for the evaluation of BiW and 4 h postbirth parameters of AC, BW, BMI, CRL, PI, RT, and CI and time to suckle, was an ANOVA using the MIXED procedure of SAS which contained the fixed effect of BiW class (LBiW, NBiW), and Sow as a random effect. Model 3, used for the evaluation of plasma FAA profiles and clinical parameters at birth and 4 h postbirth, was the same as Model 2 but included the fixed effect of Time (0, 4 h). The SLICE statement was used for performing partitioned analyses of the least-squares means for the BiW × Time interaction. Model 4, used for the pre-, post-, and during-weaning ADG, was the same as Model 2 but included the fixed effect of Suppl (Ala, Gln) and the interaction Suppl × BiW. The SLICE statement was used for performing partitioned analyses of the least-squares means for the Suppl × BiW interaction. Model 5, used for the analysis of pre-, post-, and during-weaning BW and pre- and postweaning AC, BMI, CRL, PI, and RT, was the same as Model 4 but included the fixed effect of Age or Time with repeated measurements on the same animal taken into account using the SUBJECT = animal option to define the blocks of the block-diagonal residual covariance matrix and the TYPE = SP (LIN) option to define their covariance structure. The SLICE statement was used for performing partitioned analyses of the least-squares means for the Suppl × BiW × Age/Time interaction. Model 6, used for the analysis of the differential and total white blood cell counts, GSH and GSSG concentrations and the ratio of GSH to GSSG during the weaning test was the same as Model 5, but the TYPE = VC option was used to define their covariance structure. Model 7, used for the analysis of BW, RT, differential and total white blood cell counts, GSH and GSSG concentrations, and the ratio of GSH to GSSG during the LPS test, was the same as Model 5, but the TYPE = AR (1) option was used to define their covariance structure.

For Models 1 to 7, model selection was based on Akaike’s information criterion (Ludden et al., 1994). Sow was defined as a random factor, which allowed modeling of littermates from the same sow and inference about the fixed effects, and piglet was the experimental unit. Normality of the residuals was tested using the Shapiro–Wilks test in SAS (Version 9.4; SAS Institute Incorporated, Cary, North Carolina, USA) and equality of variance assessed via the Levene’s test. When data met the assumptions of normal distribution and equal variance, differences were assessed using the Tukey–Kramer test. When data violated the assumptions of normal distribution and/or equal variance, the Wilcoxon signed-rank test was used when comparing two groups (LBiW v. NBiW, 0 v. 4 h, etc.), whilst the Kruskal–Wallis test was used when comparing four groups (LBiW-Ala, LBiW-Gln, NBiW-Ala, NBiW-Gln), followed by Dwass, Steel, Critchlow-Fligner multiple testing. Differences for all tests were considered significant at *P *≤ 0.05. Trends (0.05 > *P* < 0.10) are shown in the tables.

Model 8, used to evaluate sickness severity after LPS injection, was the GLIMMIX procedure using a Poisson model (model statement: distribution = Poisson, link = log) comprising the fixed effects of Suppl (Ala, Gln), BiW (LBiW, NBiW), Time (−15 [Basal], 360, and 720), and their interactions. The SLICE statement was used for performing partitioned analyses of the least-squares means for the Suppl × BiW × Time interaction. Model 9, used for the evaluation of activity following LPS injection, was the same as Model 8 but used a logistic model (model statement: distribution = binomial, link = logit). For both models 7 and 8 repeated measurements on the same animal were taken into account by the “RESIDUAL” keyword in the “random statement” of the GLIMMIX procedure using the autoregressive structure of the first order (type = AR (1)) for the block diagonal residual covariance matrix. Differences were assessed using the Tukey–Kramer test with *P *≤ 0.05 considered significant. Trends (0.05 > *P* < 0.10) are shown in the tables.

Plasma FAA, amino-metabolite, and AA group data collected during the weaning and the LPS tests were then analyzed with the use of MetaboAnalyst 6.0 (Pang et al., 2024). Data were log transformed (base 10) and auto scaled prior to analysis. Datasets were first analyzed by principal component analysis to lower data dimensionality and to acquire an overview by presenting trends, groupings, and classify potential outliers. Outlier samples were defined when they lay outside the 95% confidence ellipse region of the model. The separation between each experimental group (LBiW-Gln vs. LBiW-Ala, NBiW-Gln vs. NBiW-Ala, LBiW-Gln vs. NBiW-Gln, and LBiW-Ala vs. NBiW-Ala) at each Age/Time point or between each Age (27 [preweaning], 29, 30, and 32 d) or Time point (−15 [Basal], 60, 180, 360, and 720 min) within each experimental group was visualized using partial least-squares discriminant analysis (**PLS-DA**), a method that uses multivariate regression techniques to optimize separation between different groups. The quality of the PLS-DA model was verified by leave-one-out cross-validation using two performance indicators: *Q*^2^ (0.50 > *Q*^2^ < 1.0), “goodness of prediction”, or predicted variation and *R*^2^ (0.75 > *R*^2^ < 1.0), known as the goodness of fit, or explained variation, and the component (**Comp**) with the highest *Q*^2^ selected. The plasma FAA, amino-metabolites, and AA groups with *P*-values less than 0.05 based on ANOVA and variable importance in the projection (**VIP**) values higher than one were considered as discriminating metabolites.

## Results

### Zootechnical and plasma measures of experimental piglets at birth

At birth, LBiW piglets were lighter (BiW; *P* = 0.002), remained lighter at 4 h postbirth (BW; *P* = 0.002), and in addition were shorter (CRL; *P* = 0.02), thinner (AC; *P* = 0.02), and had a lower BMI (*P* < 0.001) compared with their NBiW littermates (Table 2). No difference in PI, RT, CI, or time to first suckle was observed between LBiW and NBiW littermates (Table 2). In addition, feed intake from 33 d of age until euthanasia (61 d of age) was recorded, and no difference was observed between the experimental groups (data not shown).

**Table 2.** Zootechnical parameters of male LBiW and NBiW experimental piglets, within the first 24 h of life

Parameters^1^	Time^2^	LBiW	NBiW	SE
Birthweight, kg	0	1.10^b^	1.48^a^	0.023
Bodyweight, kg	4	1.10^b^	1.50^a^	0.027
Abdominal circumference, cm	4	22.7^b^	24.8^a^	0.549
Bodymass index, cm	4	22.0^b^	24.8^a^	0.972
Crown-rump length, cm^*^	4	23.8^b^	26.0^a^	0.453
Ponderal index, cm	4	99.4	102	6.15
Rectal temperature, ^o^C	4	37.9	38.0	0.152
Time to suckle, min		50.1	40.9	7.96
Colostrum intake, g/24 h	24	392	412	57.6

^1^Values are observed means ± SE; only the largest SE is shown; *n* = 20 per BiW group.

^2^Time in h relative to birth (0 h).

^a,b^Different from NBiW piglets (*P* < 0.05).

^*^Nonparametric analysis, Wilcoxon signed-rank test.

The plasma parameters of glucose (*P* < 0.001; Table 3), ALT, AST, ALT: AST ratio, bilirubin, fructose, lactate, NEFA, total protein, and TG (*P* < 0.001; Supplementary Table 6) were affected by Time. All plasma essential AA (EAA), nine 9 of 10 nonessential AA (NEAA), seven of the nine amino-metabolites, and all the AA groups were also affected by Time (*P* < 0.001; Table 3, Supplementary Table 7). At birth (0 h), the plasma concentration of albumin (*P* = 0.04) and β-alanine (**β-Ala**; *P* = 0.03) were lower, while inositol (*P* = 0.005), Gly (*P* = 0.04), His (*P* = 0.006), Ser (*P* = 0.03), carnosine (**Car**; *P* = 0.03), and glucogenic AA (**GAA**; *P* = 0.03) were higher in LBiW compared with NBiW littermates (Table 3). At 4 h postbirth, the plasma concentration of inositol (*P* = 0.001) and Gly (*P* = 0.03) was still higher, whilst IGF-1 (*P* = 0.001) was lower in LBiW compared with NBiW littermates (Table 3). No significant difference in any of the other measured plasma clinical chemistry parameters (Supplementary Table 6) or FAA (Supplementary Table 7) was observed, between LBiW and NBiW littermates at 0 and 4 h.

**Table 3.** Concentration of plasma clinical-chemistry and amino acid parameters measured in male LBiW and NBiW experimental piglets at birth (0 h) and 4 h postbirth

Parameters^1^^,^^2^	0 h			4 h			*P* value^3^
	LBiW	NBiW	SE	LBiW	NBiW	SE	Time
Clinical-chemistry parameters							
Albumin^†^, g/L	6.75^b,h^	7.62^a^	0.257	7.58^g^	7.61	0.326	0.288
Glucose, mmol/L	2.56^f^	2.77^f^	0.296	4.72^d,e^	5.84^c,e^	0.587	<0.001
IGF-1, ng/mL	-	-	-	35.4^b^	46.4^a^	2.57	
Inositol, mmol/L	4.03^a^	2.83^b^	0.370	4.63^a^	3.58^b^	0.283	0.210
Amino acids, µmol/L							
Glycine^*^^,†^	1022^a^	840^b,f^	95.1	1111^a^	991^b,e^	51.2	0.020
Histidine	65.6^a,f^	54.9^b,f^	5.13	139^e^	151^e^	12.1	<0.001
Serine^*^	242^a,f^	205^b,f^	18.0	368^e^	342^e^	22.9	<0.001
Threonine	141^a,f^	123^b,f^	18.2	235^e^	264^e^	25.3	0.002
Tyrosine	82.4^c,f^	64.9^d,f^	12.6	259^e^	279^e^	30.2	<0.001
NEAA^*^	3211^c,f^	2834^d,f^	233	5136^e^	5017^e^	265	<0.001
Amino-metabolites							
β-Alanine^†^	7.91^b,f^	9.75^a,f^	0.701	16.0^e^	16.6^e^	1.35	<0.001
Carnosine	19.0^a^	16.7^b^	1.07	20.1^c^	17.9^d^	1.14	0.379
Hydroxyproline	91.3^e^	94.2^g^	11.2	60.1^d,f^	71.7^c,h^	6.56	0.050
AA Groups							
Glucogenic AA^*^	2096^a^	1739^b,f^	189	2307	2258^e^	122	0.020

^1^Values are observed means ± SE; only the largest SE is shown; *n* = 14–17 at 0 h per BiW group, *n* = 19–20 at 4 h per BiW group, except IGF-1, *n* = 7–9 at 4 h per BiW group.

^2^AA, Amino acids; IGF-1, Insulin-like growth factor 1.

^3^ANOVA *F* Test.

^a,b^Different from NBiW piglets within the same time point (*P* < 0.05).

^c,d^Tend to be different from NBiW piglets within the same time point (*P* < 0.10).

^e,f^Different from time points 0 to 4 h, within BiW group (*P *< 0.05).

^g,h^Tend to be different from time points 0 to 4 h, within BiW group (*P* < 0.10).

^*^Data were log transformed for the 0 h BiW group comparison.

^†^Data were log transformed for the 4 h BiW group comparison.

### Pre- and postweaning zootechnical development

During the preweaning period (1 to 28 d of age), AC, BMI, BW, and CRL were affected by BiW class and Age (*P* < 0.05), RT was affected only by Age (*P* < 0.001), and ADG was affected only by BiW (*P* < 0.05; Table 4). The LBiW piglets were always lighter than their NBiW supplementation littermates (*P* < 0.05), and the preweaning ADG was lower in LBiW-Ala than NBiW-Ala piglets (*P* = 0.03; Table 4). At 28 d of age (*P* = 0.03), NBiW-Ala were heavier than NBiW-Gln (Table 4). Abdominal circumference was smaller at 5, 7, and 28 d of age (*P* < 0.05) in LBiW-Ala compared with NBiW-Ala piglets, and at 28 d of age (*P* < 0.05) in LBiW-Gln than NBiW-Gln piglets (Table 4). At 5 to 14 and 28 d of age, LBiW-Ala were shorter (CRL; *P* < 0.05) compared with NBiW-Ala, and at 5 and 7 d of age, LBiW-Gln were shorter than NBiW-Gln (CRL; *P* < 0.05). At 21 d of age, RT was higher in LBiW-Gln than LBiW-Ala piglets (*P* = 0.05; Table 4). No differences in PI were observed.

**Table 4.** Preweaning (1–28 days of age) zootechnical development of male LBiW and NBiW piglets supplemented with alanine (Ala) or glutamine (Gln), from 1 to 12 d of age

Parameters^1^^,^^2^	Age^3^	LBiW		NBiW			*P*-value^4^	
		Ala	Gln	Ala	Gln	SE	BiW	Age
BW, kg	1	1.11^f^	1.18^f^	1.46^e^	1.55^e^	0.187	<0.001	<0.001
	5	1.70^f^	1.82^f^	2.21^e^	2.26^e^	0.187		
	7	2.07^f^	2.27^f^	2.76^e^	2.76^e^	0.187		
	14	3.90^f^	3.77^f^	4.65^c,e^	4.32^d,e^	0.187		
	21	5.41^f^	5.27^f^	6.27^e^	6.12^e^	0.187		
	28	6.58^f^	6.78^f^	7.81^a,e^	7.42^b,e^	0.187		
ADG, g/d	1-28	197^f^	203	227^e^	211	14.1	0.046	
AC, cm	5	28.0^f^	29.0	30.3^e^	30.6	0.912	<0.001	<0.001
	7	30.3^f^	30.6^h^	33.3^e^	32.7^g^	0.912		
	14	37.2^h^	37.2	39.4^g^	38.7	0.912		
	21	41.9	41.1^f^	43.0	43.5^e^	0.912		
	28	43.4^f^	43.7^f^	46.5^e^	46.5^e^	0.912		
BMI, cm^2^	5	22.1	21.3	24.7	23.8	1.40	0.035	<0.001
	7	24.1	23.8	25.3	25.6	1.40		
	14	27.9	27.0	29.7	28.8	1.44		
	21	30.6^h^	31.4	33.9^g^	32.5	1.40		
	28	34.6	35.4	35.9	34.3	1.40		
CRL, cm	5	27.0^f^	27.9^f^	29.6^e^	30.7^e^	1.00	<0.001	<0.001
	7	29.6^f^	30.2^f^	32.9^e^	32.9^e^	1.00		
	14	36.0^f^	36.0^h^	39.0^e^	38.2^g^	1.03		
	21	42.7	41.9	44.0	43.3	1.00		
	28	44.1^d,f^	46.2^c^	47.9^e^	48.0	1.00		
PI, cm^3^	5	79.2	74.2	82.5	78.3	3.59	0.613	0.622
	7	80.3	77.9	76.4	78.5	3.59		
	14	75.7	72.9	75.7	74.8	3.59		
	21	73.4	76.8	79.6	75.1	3.59		
	28	80.0	81.3	77.7	73.8	3.59		
RT, ^o^C	5	39.4	39.1	39.3	39.2	0.177	0.992	<0.001
	7	39.4	39.3	39.1	39.4	0.177		
	14	39.3	39.4	39.1	39.3	0.177		
	21	39.3^d^	39.8^c^	39.4	39.5	0.177		
	28	38.6	38.5	38.5	38.7	0.177		

^1^Values are LSM ± SE; only the largest SE is shown; *n* = 10 per BiW × Suppl group.

^2^BW, Bodyweight; ADG, Average daily gain; AC, Abdominal circumference; BMI, Bodymass index; CRL, Crown-rump length; PI, Ponderal index; RT, Rectal temperature; Suppl, Supplementation.

^3^Age, piglet age in days, with weaning at 28 days of age.

^4^ANOVA *F* Test.

^a,b^Different from Ala-supplemented piglets within BiW group (*P *< 0.05).

^c,d^Tend to be different from Ala-supplemented piglets within BiW group (*P* < 0.10).

^e,f^Different from NBiW piglets within Suppl group (*P *< 0.05).

^g,h^Tend to be different from NBiW piglets within Suppl group (*P* < 0.10).

Postweaning (35 to 61 d of age) AC was affected by BiW and Age (*P* < 0.001). Body mass index, BW, and CRL were affected by BiW and Age (*P* < 0.05), whilst PI and RT were affected only by Age (*P* < 0.05), and ADG was affected by BiW (*P* = 0.04; Supplementary Table 8). From 42 to 61 d of age, LBiW-Ala pigs were lighter than their NBiW-Ala littermates (*P* < 0.05), whilst LBiW-Gln pigs were only lighter than NBiW-Gln at 56 and 61 d of age (*P* < 0.05). Abdominal circumference was smaller at 42 to 56 d of age (*P* < 0.05) in LBiW-Ala compared with NBiW-Ala pigs, and at 49 and 61 d of age (*P* < 0.05) in LBiW-Gln than NBiW-Gln pigs. The CRL at 42 to 56 d of age was lower in LBiW-Ala compared with NBiW-Ala, and at 42, 61 d of age in LBiW-Gln than NBiW-Gln (*P* = 0.03). At 61 d of age, PI was lower in NBiW-Gln than NBiW-Ala (*P* = 0.05; Supplementary Table 8).

### Weaning BW and WBC counts

Weaning BW was affected by BiW and Age (*P* < 0.001), whilst the percentage of LYM, Band, and Segs neutrophils and the N/L ratio were affected by Age (*P* < 0.05; Table 5). The percentage of LYM was significantly (27 d of age, *P* = 0.02; Table 5) lower in LBiW-Gln than LBiW-Ala and NBiW-Gln, respectively. The percentage of Band (29 d of age, *P* = 0.05) was lower in LBiW-Gln than NBiW-Gln. There was also an increase in the N/L ratio, the Segs percentage and a decrease in the percentage of LYM, in LBiW-Ala (*P* < 0.001), NBiW-Ala (*P* < 0.05), and NBiW-Gln (*P* < 0.05) pigs. Additionally, an increase in WBC (*P* < 0.04) was observed in NBiW-Gln pigs (**Table 5**).

**Table 5.** Preweaning (27 d of age) to early postweaning (32 d of age) bodyweight development and blood leukocyte proportion of male LBiW and NBiW piglets supplemented with alanine (Ala) or glutamine (Gln), from 1 to 12 d of age

Parameters^1^^,^^2^	Age^3^	LBiW		NBiW			*P*-value^4^	
		Ala	Gln	Ala	Gln	SE	BiW	Age
Zootechnical								
BW, kg	27	6.30^d^	6.56^d,h^	7.52^c,h^	7.37^c^	0.394	<0.001	<0.001
	29	6.45^d^	6.69^f^	7.88^c^	7.31^e^	0.394		
	30	6.62^d^	6.71	7.88^c^	7.27	0.394		
	32	6.73^d^	7.30^d,g^	8.30^c,g^	8.03^c^	0.394		
Leukocytes								
LYM, %	27	67.0^a,g^	58.3^b,d^	66.9^g^	69.3^g,c^	4.34	0.211	<0.001
	29	58.0	58.1	62.5	57.9	4.52		
	30	52.9	53.8	53.3	53.5	5.61		
	32	45.2^h^	54.0	52.3^h^	50.2^h^	4.70		
MONO, %	27	2.41	2.24	2.81	1.94	0.587	0.761	0.350
	29	2.96	2.33	3.96^a^	2.54^b^	0.613		
	30	2.64	2.22	2.45	2.37	0.771		
	32	2.78	2.83	1.96	3.02	0.640		
Band, %	27	9.00	7.00	8.20	7.02^J^	1.87	0.511	0.015
	29	7.99	7.08^d^	8.16	11.8^c^	1.89		
	30	10.0	9.05	10.6	10.3	2.45		
	32	13.1	10.7	9.60	12.8^I^	1.99		
Segs, %	27	20.4^h^	22.9	21.0^h^	21.4^h^	4.13	0.269	<0.001
	29	29.5	32.4	23.4	27.8	4.28		
	30	33.0	34.9	31.7	34.1	5.21		
	32	37.4^g^	32.0	34.9^g^	33.5^g^	4.44		
N/L Ratio	27	0.485^h^	0.565	0.479^h^	0.463^h^	0.140	0.469	<0.001
	29	0.732	0.780	0.567	0.782	0.146		
	30	0.848	0.929	0.934	0.893	0.184		
	32	1.24^a,g^	0.894^b^	0.991^g^	1.00^g^	0.152		
WBC count,	27	0.929	0.870	1.10	1.04	0.300	0.219	<0.001
10^3^ cells / mL	29	1.16	1.01	1.15	1.12	0.319		
	30	1.33	1.42	1.41	1.25	0.300		
	32	1.47	1.39	1.69	1.85	0.300		

^1^Values are LSM ± SE; only the largest SE is shown; *n* = 10 per BiW × Suppl.

^2^BW, Bodyweight; LYM, Lymphocytes; MONO, Monocytes; Band, Banded neutrophils; Segs, Segmented neutrophils; Suppl, Supplementation, N/L, Neutrophil to lymphocyte; WBC, White blood cell.

^3^Age, piglet age in days, with weaning at 28 days of age.

^4^ANOVA *F* Test.

^a,b^Tend to be different from Ala-supplemented pig within BiW group (*P* < 0.10).

^c,d^Different from NBiW pigs within Suppl group (*P* < 0.05).

^e,f^Tend to be different from NBiW pigs within Suppl group (*P* < 0.10).

^g,h^Different from prior- (27 d of age) to postweaning (32 d of age) (*P *< 0.05).

^i,j^Tend to be different between prior- (27 d of age) to postweaning (32 d of age) (*P* < 0.10).

### Weaning glutathione and plasma AA profiles

The concentrations of GSH, GSSG, GSH:GSSG ratio, and total GSH were affected by Age (*P* < 0.05; Supplementary Table 9). The concentration of GSH was significantly lower in LBiW-Ala than NBiW-Ala (32 d of age; *P* = 0.05), whilst the concentration of GSSG at 27 d of age was significantly higher in NBiW-Ala than LBiW-Ala (*P* = 0.03) and NBiW-Gln pigs (*P* = 0.02). At 30 d of age, GSSG concentrations were higher in LBiW-Ala than NBiW-Ala (*P* = 0.02).

From pre- (27 d of age) to postweaning (32 d of age), there was a decrease in GSH and total GSH for all four groups (*P* < 0.05; Supplementary Table 9).

**Figure 2.**
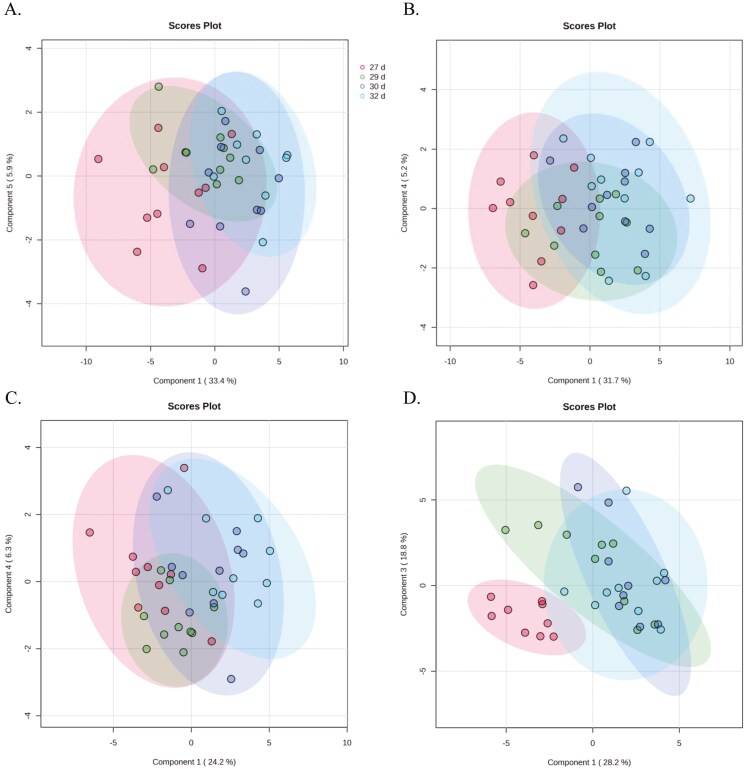
PLS-DA of plasma free amino acids and amino-metabolites between pre- (27 d of age) and post- (29, 30, 32 d of age) weaning LBiW and NBiW pigs supplemented with alanine (Ala) or glutamine (Gln) from 1 to 12 d of age. PLS-DA plot for (A) LBiW-Gln, (Measure: 5 comps; *R*^2^ = 0.95; *Q*^2^ = 0.83), (B) LBiW-Ala (Measure: 4 comps; *R*^2^ = 0.88; *Q*^2^ = 0.73), (C) NBiW-Gln (Measure: 4 comps; *R*^2^ = 0.88; *Q*^2^ = 0.67), and (D) NBiW-Ala (Measure, 3 comps; *R*^2^ = 0.82; *Q*^2^ = 0.55).

**Figure 3.**
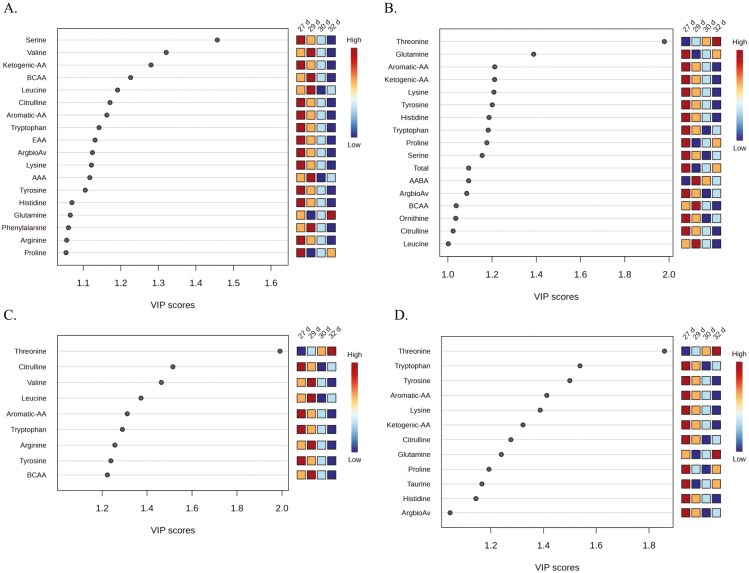
VIP generated from PLS-DA between pre- (27 d of age) and post-weaning (29, 30, 32 d of age) LBiW and NBiW pigs supplemented with alanine (Ala) or glutamine (Gln) from 1 to 12 d of age. Amino acids and amino-metabolites with a VIP score ≥ 1 and an ANOVA *P*-value ≤ 0.05 were considered as discriminating parameters and reported here for the experimental groups (A) LBiW-Gln, (B) LBiW-Ala, (C) NBiW-Gln, and (D) NBiW-Ala. The colored boxes on the right indicate the relative concentrations of the corresponding metabolite in each group under study.

Partial least squares-discrimination analysis was utilized to clarify possible discrimination of the plasma FAA, amino-metabolite, and AA group concentrations between the experimental groups and age comparisons during weaning. The result of each comparison showed that this discrimination could only be made for Age (27, 29, 30, and 32 d of age) comparisons within each experimental group; LBiW-Gln (Measure: 5 comps; *R*^2^ = 0.95; *Q*^2^ = 0.83, Figure 2A), LBiW-Ala (Measure: 4 comps; *R*^2^ = 0.88; *Q*^2^ = 0.73, Figure 2B), NBiW-Gln (Measure: 4 comps; *R*^2^ = 0.88; *Q*^2^ = 0.67, Figure 2C), and NBiW-Ala (Measure, 3 comps; *R*^2^ = 0.82; *Q*^2^ = 0.55, Figure 3D).

In LBiW-Gln piglets, the plasma FAA Ser, Val, Leu, Trp, Lys, Tyr, His, Gln, Phe, Arg, Pro, the AA groups ketogenic AA (**KAA)**, branched-chain AA (**BCAA**), aromatic AA, EAA, arginine bioavailability (**ArgbioAv**), the amino-metabolite α-aminoadipic acid (**AAA**), and the nonproteinogenic AA Citrulline (**Cit**) had a VIP ≥ 1 and *P*-value < 0.05 (Figure 3A). In LBiW-Ala piglets, the plasma FAA Thr, Gln, Lys, Tyr, His, Trp, Pro, Ser, Leu, the AA Groups aromatic AA, ketogenic AA, total AA, ArgbioAv, BCAA, the amino-metabolite α-aminobutyric acid, and the nonproteinogenic AA ornithine (**Orn**) and Cit had a VIP ≥ 1 and *P*-value < 0.05 (Figure 3B). In NBiW-Gln piglets, the plasma FAA Thr, Val, Leu, Trp, Arg, Tyr, Cit, and the AA groups aromatic AA and BCAA had a VIP ≥ 1 and *P*-value < 0.05 (Figure 3C). In NBiW-Ala piglets, the plasma FAA Thr, Trp, Tyr, Lys, Gln, Pro, His, Cit, the AA groups aromatic-AA, ketogenic AA, ArgbioAv, and the amino-metabolite Taurine (**Tau**) had a VIP ≥ 1 and *P*-value < 0.05 (Figure 3D). The *P*-values and significant ANOVA comparisons for each parameter with a VIP ≥ 1 are reported in Supplementary Table 10.

### BW, rectal temperature, WBC counts, plasma TNF-α concentrations, and sickness and activity scores after an LPS challenge

BW was affected by BiW (*P* < 0.005) and Time (*P* < 0.001), whilst RT was affected by Suppl (*P* < 0.04) and Time (*P* = 0.02; Supplementary Table 11). Throughout the experimental period, LBiW pigs remained lighter than NBiW littermates, and all pigs were lighter at 24 h (1440 min) after the LPS injection compared with the pre-injection period (*P* < 0.05; Supplementary Table 11). At 360 min, RT was higher in LBiW-Gln than LBiW-Ala (*P* < 0.001).

Lymphocyte, MONO, Band, Segs %, N/L ratio, and WBC count were affected by Time (*P* < 0.001), whilst LYM % (*P* = 0.03) and WBC count (*P* = 0.05) were affected by the interaction BiW × Suppl × Time, and BiW, respectively (Table 6). At 360-min post-LPS injection, the LYM % was lower in NBiW-Ala than LBiW-Ala pigs (*P *= 0.01). Additionally, at 360 min, the N/L ratio was higher in LBiW-Gln than NBiW-Gln, and lower in LBiW-Ala than NBiW-Ala pigs (*P* = 0.01; Table 6), and the WBC count was higher in NBiW-Gln than NBiW-Ala (*P* = 0.03) and LBiW-Gln than LBiW-Ala (*P* = 0.05) pigs. From pre- (−15 min) to 720 min post-LPS injection, there was a decrease in LYM and Segs and an increase in Band and N/L ratio (*P* < 0.001) in all four groups (Table 6). No difference in MONO or WBC count was observed.

**Table 6.** Blood leukocyte type counts and plasma TNF-α concentrations of male LBiW and NBiW pigs supplemented with alanine (Ala) or glutamine (Gln) from 1 to 12 d of age, following an intraperitoneal injection of lipopolysaccharide (LPS; 100 µg/kg bodyweight) at 55 d of age

Parameters^1^^,^^2^	Time^3^	LBiW		NBiW			*P*-values^4^		
		Ala	Gln	Ala	Gln	SE	BiW	Time	Interaction
Leukocytes									
LYM, %^*^	−15	63.2^i^	63.7^i^	63.1^i^	66.0^i^	3.19	0.445	<0.001	0.027
	360	64.4^f^	51.6	48.4^d,e^	62.3^c^	5.99			
	720	40.7^j^	38.5^j^	34.9^j^	38.2^j^	4.62			
MONO, %†	−15	1.15	2.10	1.65	1.90	0.427	0.102	0.032	0.970
	360	0.750	1.25	1.13	1.33	0.473			
	720	1.05	1.70	1.67	1.80	0.523			
Band, %*	−15	7.00^j^	5.50^j^	6.12^j^	6.55^j^	1.28	0.623	<0.001	0.560
	360	20.1	27.0	21.5	20.3	5.81			
	720	46.0^i^	46.2^i^	46.0^i^	46.1^i^	5.26			
Segs, %	−15	27.9^i^	27.7^i^	28.3^i^	24.2^i^	3.48	0.435	<0.001	0.148
	360	14.7	19.9f	18.8	12.3e	4.42			
	720	12.6^j^	13.6^j^	11.1^j^	13.9^j^	2.77			
N/L Ratio^*^	−15	0.567^j^	0.540^j^	0.578^j^	0.499^j^	0.081	0.729	<0.001	0.329
	360	0.560^f^	1.35^e^	1.16^c,e^	0.627^d,f^	0.496			
	720	1.53^i^	2.25^i^	1.87^i^	1.78^i^	0.761			
WBC count,	−15	0.925	1.38	1.13	1.18	0.317	0.047	<0.001	0.730
10^6^/mL^†^	360	0.232^b^	0.700^a^	0.531^b^	0.875^a^	0.304			
	720	0.870	1.08	1.25	1.41	0.254			
Cytokines									
TNF-α, ng/mL	−15	0.160	0.152	0.160	0.156	0.013	0.878	<0.001	0.999
	60	103^a^	78.2^b^	106^a^	76.0^b^	17.8			
	120	18.8	15.5	17.6	11.6	3.1			
	360	5.9	4.8	5.8	4.2	0.907			
	720	1.9	1.8	2.3	1.3	0.491			

^1^Values are observed means ± SE; *n* = 10 per BiW × Suppl.

^2^LYM, lymphocytes; MONO, monocytes; Band, banded neutrophils; Segs, segmented neutrophils; Suppl, Supplementation; N/L, neutrophil to lymphocyte; WBC, white blood cell count.

^3^Time, min relative to LPS injection.

^4^ANOVA *F* Test. Interaction is (BiW × Suppl × Time).

^a,b^Different from Ala-supplemented pigs within BiW group (*P *< 0.05).

^c,d^Tend to be different from Ala-supplemented pigs within BiW group (*P* < 0.10).

^e,f^Different from NBiW pigs within Suppl group (*P* < 0.05).

^i,j^Different from prior (−15 min) to 720 min post-LPS injection (*P* < 0.05).

^*^Nonparametric analysis, Kruskal–Wallis Test and Dwass, Steel, Critchlow–Fligner Post-Hoc multiple testing.

^†^Data were log transformed.

Plasma TNF-α concentrations were affected by Time (*P* < 0.001). At 60 min post-LPS, the plasma concentration of TNF-α was lower in Gln-supplemented piglets compared with Ala, irrespective of BiW (Table 6).

During the 6 h observation period following LPS administration, the activity of LBiW-Ala pigs (4.17 ± 1.44%) was lower than that of LBiW-Gln pigs (11.7 ± 2.32%; *P* = 0.01). No other differences in activity or sickness symptoms were observed.

### Glutathione and plasma AA profiles after an LPS challenge

The concentrations of GSH and GSSG were affected by Time (*P* < 0.05; Supplementary Table 12). At 360 min, the concentration of GSSG was lower in NBiW-Gln than NBiW-Ala (*P* = 0.05). From pre- (−15 min) to 720 min post-LPS injection, the ratio of GSH:GSSG (*P* = 0.05) increased in NBiW-Ala (Supplementary Table 12).

**Figure 4.**
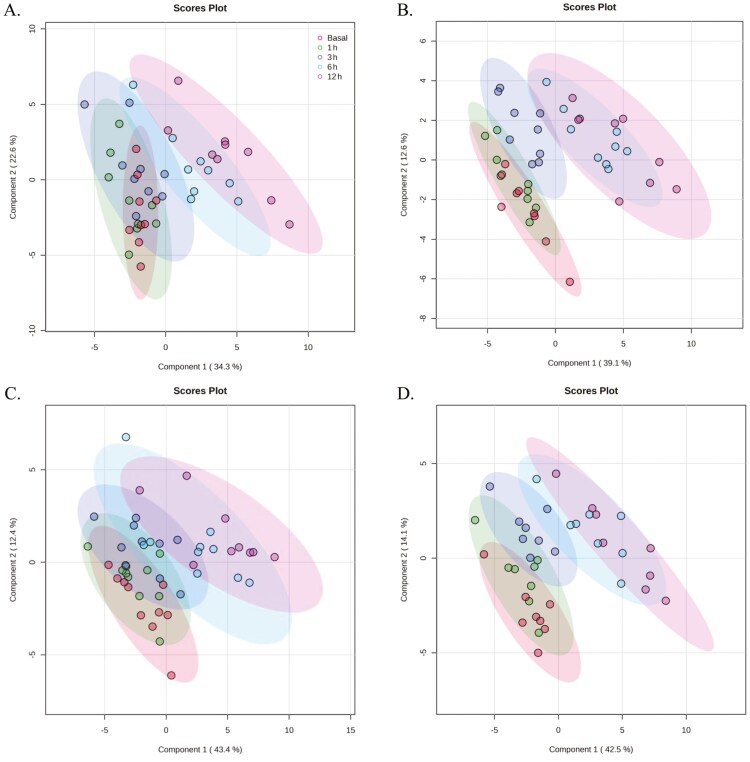
PLS-DA of plasma free amino acids and amino-metabolites between basal (−15 min) and post- (1, 3, 6, 12 h) lipopolysaccharide (100 µg/kg bodyweight) administration samples of LBiW and NBiW pigs supplemented with alanine (Ala) or glutamine (Gln) from 1 to 12 d of age. PLS-DA plot for (A) LBiW-Gln, (Measure: 5 comps; *R*^2^ = 0.93; *Q*^2^ = 0.84), (B) LBiW-Ala (Measure: 5 comps; *R*^2^ = 0.96; *Q*^2^ = 0.91), (C) NBiW-Gln (Measure: 5 comps; *R*^2^ = 0.93; *Q*^2^ = 0.84), and (D) NBiW-Ala (Measure, 5 comps; *R*^2^ = 97; *Q*^2^ = 0.92).

Partial least squares-discrimination analysis was utilized to clarify any potential discrimination of the plasma FAA between experimental group and time point comparisons. The result of each comparison showed that this discrimination could only be made for the Time (−15 [Basal], 1, 3, 6, and 12 h) comparisons within each experimental group: LBiW-Gln, (Measure: 5 comps; *R*^2^ = 0.93; *Q*^2^ = 0.84, Figure 4A), LBiW-Ala (Measure: 5 comps; *R*^2^ = 0.96; *Q*^2^ = 0.91, Figure 4B), NBiW-Gln (Measure: 5 comps; *R*^2^ = 0.93; *Q*^2^ = 0.84, Figure 4C), and NBiW-Ala (Measure, 5 comps; *R*^2^ = 0.97; *Q*^2^ = 0.92, Figure 4D).

**Figure 5.**
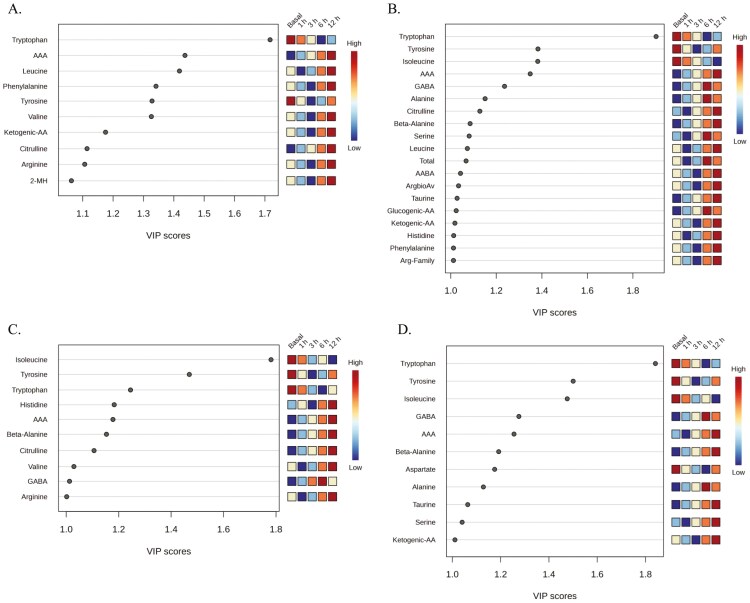
VIP generated from PLS-DA between basal (−15 min) and post- (1, 3, 6, 12 h) lipopolysaccharide (100 µg/kg bodyweight) administration samples of LBiW and NBiW pigs supplemented with alanine (Ala) or glutamine (Gln) from 1 to 12 d of age. Amino acids and amino-metabolites with a VIP score ≥ 1 and an ANOVA *P*-value ≤ 0.05 were considered as discriminating parameters and reported here for the experimental groups (**A**) LBiW-Gln, (B) LBiW-Ala, (C) NBiW-Gln, and (D) NBiW-Ala. The colored boxes on the right indicate the relative concentrations of the corresponding metabolite in each group under study.

In LBiW-Gln piglets, the plasma FAA Trp, Leu, Phe, Tyr, Val, Arg, Cit, the AA group ketogenic AA, and the amino-metabolites AAA and 3-methylhistidine had a VIP ≥ 1 and *P*-value < 0.05 (Figure 5A), whilst in LBiW-Ala piglets, the plasma FAA Trp, Tyr, Iso, Ala, Ser, Leu, His, Phe, Cit, the AA groups total AA, ArgbioAv, glucogenic AA, ketogenic AA, Arginine-Family AA, and the amino-metabolites AAA, γ-aminobutyric acid (**GABA**), β-Ala, α-aminobutyric acid, and Tau had a VIP ≥ 1 and *P*-value < 0.05 (Figure 5B). In NBiW-Gln piglets, the plasma FAA Ile, Tyr, Trp, His, Val, Arg, Cit, and the amino-metabolites AAA, β-Ala, and GABA, had a VIP ≥ 1 and *P*-value < 0.05 (Figure 5C), whilst in NBiW-Ala piglets, the plasma FAA Trp, Tyr, Ile, aspartate, Ala, Ser, the AA group ketogenic AA, and the amino-metabolites GABA, AAA, β-Ala, and Tau had a VIP ≥ 1 and *P*-value < 0.05 (Figure 5D). The *P*-values and significant ANOVA comparisons for each parameter with a VIP ≥ 1 are reported in Supplementary Table 13.

## Discussion

We have previously reported that suckling LBiW pigs supplemented with Gln were heavier than their Ala supplemented LBiW littermates, from 11 to 21 d of age (Schregel et al., 2024), and had lower TG and higher carnosine in plasma at 5 d of age and higher milk intake at 12 d of age (Li et al., 2022). Together these results indicated that Gln supplementation alters LBiW piglet metabolism during and improved BW after, the supplementation period, compared with LBiW pigs supplemented with Ala. It has been reported that LBiW pigs do not do as well as NBiW pigs during weaning (Zotti et al., 2017) and in response to pathogenic infection. Therefore, this study was conducted to determine if there was a carryover effect of Gln supplementation in LBiW pigs, on immune markers of stress during weaning (27 to 32 d of age) and an LPS challenge (55 d of age).

### Low birthweight piglets

In the present study, we show that LBiW piglets selected at birth were not just lighter, shorter, and thinner than their NBiW littermates, but that the concentration of plasma inositol was higher at 0 and 4 h postbirth in LBiW compared with NBiW piglets. Higher plasma inositol concentrations at birth have been proposed as a biomarker of IUGR in infants and pigs (Nissen et al., 2011), indicating that the LBiW animals selected for this study were less mature at birth than their NBiW littermates. Interestingly in adult humans, higher blood and urine inositol levels have been linked to the development of insulin resistance, and higher plasma levels of the dipeptide carnosine, which were observed at birth, are proposed as a mechanism to counteract insulin resistance (Jukic et al., 2021). The higher plasma Car observed in this study may also explain the lower concentration of β-Ala which is the rate-limiting factor in the synthesis of Car (Jukic et al., 2021), and suggests that LBiW piglets may synthesize and release into the circulation additional Car to counteract the development of insulin resistance. Additionally, it has been reported that there is a positive relationship between fetal weight and albumin levels (Stone, 1981) and potentially explains why the plasma albumin concentration at birth was lower in LBiW than NBiW piglets from this study. Consistent with previous studies (Morise et al., 2008), we report that plasma concentrations of IGF-1 are lower in LBiW than NBiW piglets, which has been linked to impaired muscle development (Chen et al., 2017) and slower growth during the postnatal period (He et al., 2016).

The abundance of serine hydroxymethyl transferase, the enzyme required to convert Ser to Gly, is lower in LBiW compared with NBiW piglets (Hu, 2017), and when exposed to sows milk, which does not contain sufficient Gly to meet maximal neonatal pig growth requirements (Wang et al., 2014), the neonatal piglet synthesizes Gly from hydroxyproline (Hu, 2017). Therefore, in LBiW compared with NBiW piglets, the higher plasma Ser concentration at birth maybe a consequence of lower serine hydroxymethyl transferase abundance, and the reduced hydroxyproline concentrations at 4 h postbirth, an attempt by LBiW piglets to meet Gly needs for growth. We speculate that the higher Gly concentrations at birth and 4 h postbirth suggest that LBiW piglets may not be able to fully utilize Gly for growth requirements. The higher levels of plasma GAA at birth, which is the sum total of Ser, Gly, and Ala, can be explained by the significantly higher levels Ser and Gly in LBiW compared with NBiW pigs.

It has also been proposed that LBiW piglets compared with NBiW, take longer to make their first suckle, and/or their colostrum intake in the first 24 h of life is lower (Vodolazska et al., 2023). In this, and a companion study (Li et al., 2022), we show that there was no significant difference in estimated colostrum intake and time to first suckle between LBiW and NBiW piglets. This may explain why at 4 h postbirth, no difference was observed between LBiW and NBiW piglets in plasma factors which can only be obtained from the colostrum, such as immunoglobulins or indispensable AA.

### Pre- and postweaning growth

Across multiple studies, the association of Gln and Gln-dipeptide supplementation with improved pre- and postweaning BW and growth has been inconsistent (Yi et al., 2005; Haynes et al., 2009; Jiang et al., 2009; Wu et al., 2011). The few pre-weaning direct supplementation studies that have been conducted in suckling piglets have shown improved ADG, but no effect on BW (Haynes et al., 2009; Wu et al., 2011), whilst Gln supplementation given together with creep feed had inconsistent effects on both ADG and BW or no effect on BW (Cabrera et al., 2013; Arnaud et al., 2024). Postweaning, some studies have reported improved ADG only after an *E. coli* K88+ (Yi et al., 2005; Hsu et al., 2012) or LPS (Jiang et al., 2009) challenge, with no report on BW. Two postweaning studies where BW was reported showed no difference, but improved ADG (Zou et al., 2019; Luise et al., 2022). It should be noted that none of the aforementioned studies investigated the effect of Gln supplementation on LBiW piglets.

We have previously published that LBiW piglets supplemented with Gln from 1 to 12 d of age was associated with higher BW from 11 to 21 d of age, compared with LBiW-piglets supplemented with Ala whilst remaining lighter than NBiW piglets supplemented with Gln (Schregel et al., 2024). These observations suggested a carryover effect of Gln compared with Ala in LBiW piglets. In the current study, we could not confirm our earlier observation of a growth advantage of LBiW-Gln vs. LBiW-Ala piglets and we also observed no catchup growth of LBiW compared with NBiW piglets supplemented with Gln, during and postsupplementation (Schulze Holthausen et al., 2022). This was an unexpected result, which we hypothesized may have been due to the BW assignment at 24 h postbirth leading to heavier Ala piglets in the LBiW group, and thus, negated any potential increase in BW gain by Gln littermates. The 24 h postbirth BW was not significantly different, between the Ala and Gln supplemented LBiW piglets in this study, or the two companion studies (Li et al., 2022; Schregel et al., 2024). Therefore, these results cannot explain why no difference in BW could be observed in LBiW piglets supplemented with Gln compared with Ala.

Postweaning, changes in zootechnical parameters from 35 to 61 d of age, and plasma metabolites and AA at 61 d of age were observed in LBiW pigs supplemented with Gln compared with their Ala supplementation and NBiW littermates. However, the changes were not consistent and thus, we conclude that Gln supplementation from 1 to 12 d of age to LBiW pigs does not appear to change postweaning performance.

### Glutamine supplementation and weaning stress

Conventional weaning of pigs has been shown to negatively affect growth, intestinal development, AA metabolism, and antioxidative defense, feed intake, and health (Montagne et al., 2007; Campbell et al., 2013; Xiong et al., 2019; Blavi et al., 2021). Glutamine supplementation studies during- and postweaning have reported increased feed efficiency, improved growth performance and immune response to pathogenic challenge, prevention of jejunal atrophy, and increased circulatory and intestinal GSH concentrations (Wang et al., 2008; Wu et al., 2011; Xiong et al., 2019; Li et al., 2024). To date, we could find no studies examining the effect of Gln on these parameters when administered to piglets in the suckling phase. Here, we show that Gln supplementation from 1 to 12 d of age did not improve weaning BW, FCR, RBC GSH status, or results in significant differences in plasma AA profiles of LBiW or NBiW piglets compared with Ala, but appears to have improved the stress response/health of LBiW piglets supplemented with Gln.

The N/L ratio is a useful and simple measure of how healthy or “stressed” a pig is, and it has been used to assess the impact of weaning environment and age (Puppe et al., 1997; Salak-Johnson and Webb, 2018). Previous studies have reported that at day 1 after weaning (29 d in the current study), there is a spike in the N/L ratio which declines back to preweaning levels at day 4 (32 d of age in the current study) (Puppe et al., 1997) and day 7 postweaning (Salak-Johnson and Webb, 2018). In agreement with both studies, we observed the same 29 d of age N/L ratio spike in all groups. However, only the LBiW pigs supplemented with Gln had declined to preweaning N/L ratio levels by 32 d of age, whilst the N/L ratio of the other three experimental groups was still increasing, indicative of prolonged weaning stress. Thus, it would appear that LBiW pigs supplemented with Gln had adapted to their new postweaning housing environment and social order before the other three groups. Why this occurred may be due to a blunted cortisol response in LBiW pigs supplemented with Gln (Moscovice et al., 2022).

The N/L shift is proposed to be triggered by a rise in cortisol which simultaneously increases LYM and decreases neutrophils (Wirthgen et al., 2018). Whilst cortisol was not measured in the current study, we do show that there is a significant decrease in LYM and increase in Segs neutrophils, from 27 to 32 d of age, in all groups except LBiW pigs supplemented with Gln. This result is a strong indication that the cortisol surge observed in weaning piglets (Moscovice et al., 2022) may play a role in the stress observed from the pre- (27 d of age) to early post- (32 d of age) weaning period observed in the current study. It has been reported that Gln supplementation during weaning can attenuate or blunt the cortisol surge (Zhou et al., 2006), by a mechanism that may be linked to increased availability of Gln in supplemented pigs. In vitro studies using intestinal tissue harvested from weanling pigs have shown that cortisol increases the metabolism of Gln into Cit (Flynn and Wu, 1997; Wu et al., 2000). Therefore, supplemental Gln may prevent the depletion of Gln by cortisol, sparing it for other processes required during weaning. However, in the current study, Gln supplementation ceased at 12 d of age, and companion studies report no difference in longissimus muscle (Zhao et al., 2020), jejunum, and plasma concentrations (Schregel et al., 2024) of Gln and Gln metabolites (Arg, Asp, Cit, Glu, Orn, Pro) at 26 d of age. Thus, it is unlikely LBiW pigs supplemented with Gln and in this study have a larger pool of Gln prior to weaning.

It should be noted that at 27 d of age, LBiW pigs supplemented with Gln have a lower LYM % compared with supplementation and BiW controls. However, when compared with other studies investigating the effect of weaning at 28 d of age on innate immune status, the lymphocyte percentage in LBiW pigs supplemented with Gln in the current study was lower than in two studies (Niekamp et al., 2007; Salak-Johnson and Webb, 2018), and comparable to another (Sutherland et al., 2009). Thus, we can report that, whilst the LYM % value in LBiW pigs supplemented with Gln from the current study is lower when compared with supplementation and BiW controls, it falls into the ranges observed in other studies.

In agreement with other weaning studies, conducted in pigs, we observed a decrease in RBC GSH and total GSH concentrations and no effect on the ratio of GSH:GSSG, from the pre- (27 d of age) to early postweaning (32 d of age) period, in all four experimental groups (Degroote et al., 2019, 2020). Glutathione plays a critical role in protecting cells from oxidative damage where it is converted into GSSG, and the ratio of GSH to GSSG is used as an indicator of oxidative stress (Degroote et al., 2020; He et al., 2024). Thus, whilst weaning was associated with lower levels of GSH, it did not appear to be associated with increased oxidative stress in the RBCs of the pigs used in this study. The GSH molecule is composed of the AA Glu, Gly, and Cys, and GSH synthesis depends on the availability of Cys and Gly which are both proposed to be rate limiting factors (McCarty et al., 2018). Weaning has been reported to affect plasma AA profiles in pigs (Dobrowolski and Śliwa, 2008; Gachman et al., 2023); therefore, it was hypothesized that weaning may impact the availability of these three AA, potentially leading to the observed reduction in RBC GSH concentrations. The concentrations of plasma Cys, Gly, and Glu were unaffected from the pre- (27 d of age) to early postweaning (32 d of age) period; thus, it remains unexplained why RBC GSH concentrations decrease in response to weaning.

Assessment of the plasma AA and amino-metabolite profiles at each weaning day using PLS-DA showed that there was no significant separation between the experimental groups, indicating that Gln supplementation from 1 to 12 d of age to LBiW or NBiW pigs does not appear to change the plasma AA and amino-metabolite profiles during weaning. Therefore, a second analysis was performed assessing the FAA and amino-metabolite profiles between each of the weaning days (pre- [27 d of age], postweaning [29, 30, and 32 d of age]), within the experimental groups. There was significant separation between the different days around weaning, and variables with a VIP score ≥ 1 and an ANOVA *P*-value ≤ 0.05 were identified as contributing to the separation between weaning days. In all four experimental groups, the aromatic AA group, the individual aromatic AA Trp and Tyr, and the nonproteinogenic AA Cit were variables that significantly contributed to the separation between the weaning day groups. In each experimental group, there was a decrease in these variables from pre- to postweaning, which has been observed in several studies investigating the effect of weaning stress on plasma AA profiles (Berkeveld et al., 2008; Metzler-Zebeli et al., 2023; van Kempen et al., 2023). It has been reported that during weaning, the liver concentrations of aromatic AA (Phe, Trp and Tyr) in pigs decrease, due to increased Trp catabolism and partitioning of Phe away from Tyr synthesis to the synthesis of acute phase proteins (Huang et al., 2020). Although the liver of the animals used in this study was not assessed, this would explain why plasma concentrations of aromatic AA decreased from the pre- to postweaning phases. Plasma Cit has been shown to decrease during weaning where it had been proposed as a marker for monitoring postweaning intestinal function and mass (Berkeveld et al., 2008; Soraci et al., 2023). An interesting result from the PLS-DA analysis was that the top VIP for three (LBiW-Ala, NBiW-Gln, and NBiW-Ala) experimental groups was Thr, one of the few AA whose concentration increased from pre- to postweaning. This contradicts previous weaning studies in pigs that have reported decreased plasma Thr concentrations (Dobrowolski and Śliwa, 2008; Wang et al., 2021; Metzler-Zebeli et al., 2023) from the pre- to postweaning transition. Threonine is an essential AA and thus can only be obtained from the diet or from the breakdown of Thr containing proteins. Regardless of the source, this would suggest that Thr metabolism is negatively impacted by weaning in the animals used in this study. Threonine normally has a high rate of extraction by the intestine, with a first pass metabolism of 61% of the enteral input (Stoll et al., 1998), due to its importance in the synthesis of mucins, which have a composition of 13% to 26% Thr (Le Floc’h et al., 2012). Altogether, with the reduced plasma Cit concentration, these results may suggest that during weaning, these pigs have a smaller intestinal mass, and thus a lower requirement for Thr, leading to increased plasma concentrations.

### Glutamine supplementation and an LPS challenge

In the current study, supplementation of LBiW pigs with Gln from age 1 to 12 d of age did not affect RT, BW, or the RBC concentrations of glutathione, following an LPS injection at 55 d of age (43 d of age after the last oral dose of Gln was given), compared with supplementation and BiW controls.

In line with other porcine studies investigating the effect of an LPS challenge on circulating WBC, there was an increase in the N/L ratio from pre- to post- (720 min) LPS injection, driven by increased BAND and decreased LYM % and Segs neutrophils (Kluess et al., 2015; Tesch et al., 2015; Wirthgen et al., 2018). It has also been reported that following an LPS challenge, there is a decrease in WBC (Williams et al., 2009; Terenina et al., 2017) as they redistribute from the circulation and into the tissues. The redistribution is regulated in part by increased secretion of pro-inflammatory cytokines such as TNF-α, IL-6, and IL-1β (Ince et al., 2018). In the current study, the initial redistribution of WBCs in response to LPS (pre 360 min) was significantly higher in LBiW compared with NBiW pigs, independent of supplementation, suggesting higher secretion of pro-inflammatory cytokines, such as TNF-α, IL-1β, and IL-6 in LBiW piglets. The cytokine TNF-α was measured in this study and results showed that its peak at 1 h post-LPS administration was lower in Gln compared with Ala-supplemented piglets, independent of birthweight. Previous studies have shown that in response to an LPS challenge, in vitro production of IL-1β by peripheral blood mononuclear cells and plasma IL-6 concentrations 3 h post-LPS injection are lower in LBiW compared with NBiW pigs. However, no difference in TNF-α concentrations, between LBiW and NBiW groups, was observed in either study, suggesting that TNF-α may respond differently to IL-1β and IL-6 (Tuchscherer et al., 2012; Amdi et al., 2020). Studies investigating the effect of Gln-supplementation on ameliorating the effect of LPS have been conducted in weaned pigs. It was reported that Gln had no effect on plasma concentrations of TNF-α or IL-1β (Hsu et al., 2012) post LPS-administration but decreased TNF-α similar to what was observed in the current study (He et al., 2022).

A PLS-DA analysis was used to assess the plasma AA and amino-metabolite profiles at each time point, between the experimental groups, with results showing that there was no significant separation between the experimental groups, indicating that Gln supplementation from 1 to 12 d of age to LBiW or NBiW pigs does not appear to change the plasma AA and amino-metabolite profiles post-LPS administration. A subsequent analysis was performed assessing the FAA and amino-metabolite profiles between each of the LPS test time points (pre- [−15 min] and 1, 3, 6, and 12 h postadministration), within the experimental groups. There was significant separation between the different LPS test time points, and variables with a VIP score ≥ 1 and an ANOVA *P*-value ≤ 0.05 were identified as contributing to the separation. Similar to the weaning PLS-DA analysis, the aromatic-AA Trp and Tyr were variables that significantly contributed to the separation between the LPS test time points, for all experimental groups. This suggests a similar mechanism of partitioning aromatic-AA toward the synthesis of acute phase proteins, as observed during the weaning test, and reported in other LPS-administration studies conducted in pigs (Dritz et al., 1996; Williams et al., 2009). An amino-metabolite that also contributed to the separation between the LPS test time points for all experimental groups was the lysine degradation product AAA. The plasma concentration of AAA increased during the LPS test, and it has been reported that pigs exposed to the mycotoxin deoxynivalenol and LPS have decreased concentrations of liver AAA (Danicke et al., 2020). Altogether these results may suggest increased Lys metabolism in response to LPS administration, and export of AAA into the plasma.

## Conclusions

The LBiW piglets measured in this study had zootechnical and metabolic markers associated with impaired development, and supplementation with glutamine from 1 to 12 d of age moderately affected immune markers of weaning stress in LBiW pigs, suggesting better postweaning environment adaptation than birthweight and supplementation control piglets. Post-LPS challenge, a lower plasma TNF-α peak was observed in Gln-supplemented LBiW and NBiW piglets, indicating a lower proinflammatory response. Lastly, Gln supplementation did not improve LBiW pig BW, contrary to two published companion studies. There is no current explanation for these competing results in regards to BW and warrants further investigation.

## Supplementary Material

skaf296_suppl_Supplementary_Material

## Data Availability

The original contributions presented in this study are included in the article/Supplementary Material; further inquiries can be directed to the corresponding author.

## Abbreviations

AAA: α-aminoadipic acid
α-AA: amino acid
AC: abdominal circumference
Ala: alanine
ALT: alanine aminotransferase
ArgbioAv: arginine bioavailability
AST: aspartate aminotransferase
Band: banded neutrophils
β-Ala: β-Alanine
BCAA: branched-chain AA
BiW: birthweight
BMI: body mass index
BW: bodyweight
Car: carnosine
CI: colostrum intake
Cit: citrulline
Comp: component
CP: crude protein
CRL: crown-rump length
EAA: essential AA
FAA: free amino acids
FBN: research institute for farm animal biology
GAA: glucogenic AA
GABA: γ-aminobutyric acid
Gln: glutamine
GSH: reduced glutathione
GSSG: oxidized glutathione
HyP: hypotaurine
Ig: immunoglobulin
IGF-1: insulin-like growth factor-1
IGFBP-2-5: insulin-like growth factor binding protein-(2,3,4,5)
KAA: ketogenic AA
LBiW: low birthweight
LPS: lipopolysaccharide
LYM: lymphocytes
Orn: ornithine
NEAA: non-essential AA
NEFA: non-esterified fatty acids
ME: metabolizable energy
MONO: monocytes
NBiW: normal birthweight
N/L: neutrophil to lymphocyte ratio
PI: ponderal index
RT: rectal temperature
Segs: segmented neutrophils
Suppl: supplementation group
Tau: taurine
TNF-α: tumor necrosis factor-α
TG: triglycerides
VIP: variable importance in the projection
WBC: white blood cells
