# Supplementary material for: Association of glutamine supplementation during the early suckling period with growth, weaning, and lipopolysaccharide induced stress in low birthweight piglets
Source: J Anim Sci. 2025 Aug 30;103:skaf296. doi: 10.1093/jas/skaf296 (PMC12448690; doi:10.1093/jas/skaf296)
Supplement: skaf296_suppl_Supplementary_Material [file skaf296_suppl_supplementary_material.docx]

Association of glutamine supplementation during the early suckling period with growth, weaning and lipopolysaccharide induced stress in low birthweight piglets

By Sciascia et al. “Online Supplementary Material”

**Supplementary Table 1:** Litter characteristics at birth of sows selected for the study.

| Parameter^1^ | Values^2^ |
| --- | --- |
| *Number of litters* | 14 |
| *Total number of piglets* | 215 |
| *Total number of females* | 110 |
| *Total number of males* | 105 |
| Litter size at birth, *per litter* | 15.5 ± 0.67 |
| Stillborn, *total* | 9 |
| Live born females, *per litter* | 7.64 ± 0.55 |
| Live born males, *per litter* | 7.07 ± 0.64 |
| Total live born, *per litter* | 14.7 ± 0.72 |
| Bodyweight, *kg* |  |
| Litter, *per litter* | 19.6 ± 0.08 |
| Piglet, *per litter* | 1.26 ± 0.02 |
| Female, *per litter* | 1.23 ± 0.03 |
| Males, *per litter* | 1.29 ± 0.03 |
| Live born, *per litter* | 1.27 ± 0.02 |
| Intra-litter variability, *kg*^3^ | 0.26 ± 0.01 |

^1^Piglets born alive or dead (Mummies not included).

^2^Values are observed means ± SE.

^3^Intra-litter variability of birth weight was calculated as square root of the pooled litter variance of male and female piglets within each litter.

**Supplementary Table 2:** Feeding schedule of experimental gilts.

| Experimental period | Morning Feeding (0730 h), *kg* | Afternoon feeding (1400 h), *kg* |
| --- | --- | --- |
| Pregnancy, *days post-insemination* |  |  |
| 0 (insemination) until 108 | 1.80 | 1.80 |
| 109 - 112 | 1.80 | 1.80 |
| 113 | 1.25 | 1.25 |
| 114 | 1.00 | 1.00 |
| Lactation, *days* |  |  |
| 0 (Farrowing) | 0.50 | 0.50 |
| 1 | 0.50 | 1.00 |
| 2 | 1.00 | 1.00 |
| 3 | 1.00 | 1.50 |
| 4 | 1.50 | 1.50 |
| 5 | 1.50 | 2.00 |
| 6 | 2.00 | 2.00 |
| 7 | 2.00 | 2.50 |
| 8 | 2.50 | 2.50 |
| 9 | 2.50 | 3.00 |
| 10 | 3.00 | 3.00 |
| 11 | 3.00 | 3.50 |
| 12 | 3.50 | 3.50 |
| 13 | 3.50 | 4.00 |
| 14-27 | 4.00 | 4.00 |

**Supplementary Table 3:** Colostrum composition and immunoglobulins concentrations of experimental sows selected for the study.

|  |  | 2 h | |  | 24 h | |  | *P*-value^2^ |
| --- | --- | --- | --- | --- | --- | --- | --- | --- |
| Parameter^1^ | Teat pair | OM | SE |  | OM | SE |  | Time |
| Proximate composition, % |  |  |  |  |  |  |  |  |
| Dry matter | 1, 2 | 27.8^c^ | 1.20 |  | 25.7^d^ | 0.618 |  | 0.001 |
|  | 6, 7 | 28.5^a^ | 1.34 |  | 24.8^b^ | 1.04 |  |  |
| Crude protein | 1, 2 | 15.2^a^ | 0.603 |  | 8.38^b^ | 0.659 |  | *<0.001* |
|  | 6, 7 | 15.6^a^ | 0.526 |  | 8.86^b^ | 0.870 |  |  |
| Crude fat | 1, 2 | 7.11^b^ | 0.733 |  | 10.9^a^ | 0.627 |  | *<0.001* |
|  | 6, 7 | 7.51^b^ | 0.833 |  | 9.93^a^ | 0.638 |  |  |
| Lactose | 1, 2 | 1.66^b^ | 0.153 |  | 2.92^a^ | 0.118 |  | 0.570 |
|  | 6, 7 | 1.61^b^ | 0.152 |  | 2.96^a^ | 0.160 |  |  |
| Immunoglobulins, mg/mL |  |  |  |  |  |  |  |  |
| IgA | 1, 2 | 12.8^a^ | 0.751 |  | 6.22^b^ | 0.983 |  | *<0.001* |
|  | 6, 7 | 13.4^a^ | 0.779 |  | 6.69^b^ | 1.08 |  |  |
| IgG* | 1, 2 | 41.5^a^ | 3.97 |  | 12.0^b^ | 1.91 |  | *<0.001* |
|  | 6, 7 | 42.4^a^ | 4.57 |  | 14.4^b^ | 2.66 |  |  |
| IgM^ƚ^ | 1, 2 | 7.68^a^ | 0.537 |  | 4.28^b^ | 0.360 |  | *<0.001* |
|  | 6, 7 | 7.81^a^ | 0.665 |  | 4.37^b^ | 0.454 |  |  |

^1^Values are observed means (OM) ± SE; Proximate composition: *n* = 11 sows; Immunoglobulins: *n* = 12 sows.

^2^ANOVA *F* Test.

^a,b^Different from 2 h (*P* < 0.05).

^e,f^Tend to be different from 2 h (*P* < 0.10).

^ƚ^Data was log transformed.

*Non parametric analysis, Wilcoxon signed-rank test.

The colostrum components of dry matter (*P* = 0.001), IgA, IgG, IgM, crude protein and fat (*P* < 0.001) were affected by Time. There was a significant (teat pair 6, 7: 28.5 v. 24.8%; *P* = 0.002) and a trending (teat pair 1, 2: 27.8 v. 25.7%; *P* = 0.06) decrease in colostrum dry matter content and crude protein (teat pair 1, 2: 15.2 v. 8.4%; teat pair 6, 7: 15.6 v. 8.86; *P* < 0.001) and an increase in crude fat (teat pair 1, 2: 7.11 v. 10.9%; *P* < 0.001; teat pair 6, 7: 7.51 v. 9.93; *P* = 0.003) and lactose (teat pair 1, 2: 1.66 v. 2.92%; *P* < 0.001; teat pair 6, 7: 1.61 v. 2.96; *P* < 0.001), from 2 to 24 h, respectively. There was a significant decrease in colostrum IgA (teat pair 1, 2: 12.8 v. 6.22 mg/mL; *P* < 0.001; teat pair 6, 7: 13.4 v. 6.69; *P* < 0.001), IgG (teat pair 1, 2: 41.5 v. 12.0 mg/mL; *P* < 0.001; teat pair 6, 7: 42.4 v. 14.4; *P* < 0.001) and IgM (teat pair 1, 2: 7.68 v. 4.28 mg/mL; *P* < 0.001; teat pair 6, 7: 7.81 v. 4.37; *P* < 0.001) from 2 to 24, respectively.

**Supplementary Table 4:** Piglet losses and causes thereof in low (LBiW) and normal (NBiW) birthweight piglets supplemented with alanine (Ala) or glutamine (Gln).

| Exclusion criteria | Exclusion |  | Number of piglets | | | | | | |
| --- | --- | --- | --- | --- | --- | --- | --- | --- | --- |
|  | Code |  | LBiW | NBiW |  | LBiW-Gln | NBiW-Gln | LBiW-Ala | NBiW-Ala |
| Bodyweight loss, > 2 consecutive days | 1 |  |  |  |  | 2 | 3 | 3 | 2 |
| Sickness Symptoms |  |  |  |  |  |  |  |  |  |
| Diarrhoea and coughing | 2 |  |  |  |  | 4 | 4 | 2 |  |
| Lack of mobility | 3 |  |  |  |  | 1 |  |  | 1 |
| Died during the trial | 4 |  |  |  |  |  |  |  |  |
| Pre-supplementation (< 24 h after birth) |  |  |  |  |  |  |  |  |  |
| Squashed by the sow |  |  | 2 | 2 |  |  |  |  |  |
| Post-supplementation |  |  |  |  |  |  |  |  |  |
| Squashed by the sow |  |  |  |  |  | 2 | 1 | 4 | 1 |
| Unknown |  |  |  |  |  | 3 |  | 2 |  |
| Died during LPS test |  |  |  |  |  | 1 |  | 2 | 2 |
| Not enough experimental piglets^1^ | 5 |  |  |  |  |  |  | 1 | 1 |
| Experimental littermate removed^2^ | 6 |  |  |  |  | 5 | 7 |  | 6 |
|  | Subtotal |  | 2 | 2 |  | 18 | 15 | 14 | 13 |
| Total number of animals excluded from the study | |  | 64 |  |  |  |  |  |  |

^1^Piglets from other experimental groups were removed from the study due to death or sickness, leaving insufficient numbers of piglets to continue in that experimental block.

^2^Experimental littermate was removed due to sickness or death, and the remaining piglet continued in the study but was assigned to be a “social” piglet at the time of slaughter.

**Supplementary Table 5:** Plasma clinical chemistry parameters of pigs with low (LBiW) and normal (NBiW) birthweight (BiW) at 61 days of age, supplemented with alanine (Ala) or glutamine (Gln) from 1 to 12 days of age.

| Parameters^1,2^ | LBiW | |  |  | NBiW | |  | *P*-value^3^ |
| --- | --- | --- | --- | --- | --- | --- | --- | --- |
|  | Ala | Gln | SE |  | Ala | Gln | SE | BiW |
| Albumin, g/L | 27.7 | 29.9 | 1.53 |  | 28.3 | 28.5 | 1.36 | 0.717 |
| ALT, U/L^ƚ^ | 51.0 | 57.6 | 5.22 |  | 49.5 | 50.5 | 3.89 | 0.199 |
| AST, U/L^ƚ^ | 75.4 | 84.6^e^ | 16.1 |  | 58.9 | 62.6^f^ | 4.07 | 0.039 |
| Bilirubin, µmol/L | 1.78 | 1.75 | 0.361 |  | 1.55 | 1.22 | 0.256 | 0.209 |
| Cholesterol, mmol/L* | 1.69 | 2.00 | 0.217 |  | 1.82 | 1.78 | 0.091 | 0.681 |
| Glucose, mmol/L | 7.31 | 7.20 | 0.244 |  | 7.34 | 7.73 | 0.290 | 0.223 |
|  |  |  |  |  |  |  |  |  |
| Lactate, mmol/L | 3.22^b,f^ | 5.58^a^ | 1.05 |  | 5.19^e^ | 5.68 | 1.00 | 0.202 |
| NEFA, µmol/L | 283 | 331 | 81.3 |  | 281 | 227 | 48.8 | 0.341 |
| Total protein, g/L^*,#^ | 44.2^b,d^ | 48.1^a^ | 1.29 |  | 50.8^c^ | 48.3 | 2.59 | 0.091 |
| Triglycerides, mmol/L | 0.400 | 0.432 | 0.039 |  | 0.371 | 0.457 | 0.045 | 0.952 |
| Urea, mmol/L | 1.57 | 2.18 | 0.567 |  | 2.38 | 1.85 | 0.877 | 0.563 |

^1^Values are observed means ± SE; only the largest SE is shown; *n* = 10 per BiW x Suppl group.

^2^ALT, Alanine aminotransferase; AST, Aspartate aminotransferase; NEFA, Non-esterified fatty acids; Suppl, Supplementation.

^3^ANOVA *F* Test.

^ƚ^Data was log transformed.

*Non-parametric analysis, Kruskal-Wallis-Test and ^#^Dwass, Steel, Critchlow-Fligner Post-Hoc multiple testing^.^

^a,b^Tend to be different from Ala-supplemented pigs within BiW group (*P* < 0.10).

^c,d^Different from NBiW pigs within Suppl group (*P* < 0.05).

^e,f^Tend to be different from NBiW pigs within Suppl group (*P*< 0.10).

Plasma AST (*P* = 0.04) was significantly affected by BiW. A Kruskal-Wallis test showed that there was a significant difference of means for total protein between all four groups (H = 9.03, *P* = 0.03). To assess pairwise comparisons the Dwass, Steel, Critchlow-Fligner multiple comparison (post-hoc) test was conducted, which showed total protein was (z = 2.65, *P* = 0.04) lower in LBiW-Ala than NBiW-Ala.

**Supplementary Table 6:** Plasma clinical chemistry parameters and immunoglobulin (Ig) concentrations of low (LBiW) and normal (NBiW) birthweight (BiW) experimental piglets at birth (0 h) and 4 h post-birth.

| Parameters^1,2^ | 0 h | |  | 4 h | |  |  | *P*-value^3^ |
| --- | --- | --- | --- | --- | --- | --- | --- | --- |
|  | LBiW | NBiW | SE | LBiW | NBiW | SE |  | Time |
| Clinical-chemistry |  |  |  |  |  |  |  |  |
| ALT, U/L^*,ƚ^ | 11.1^b^ | 12.2^b^ | 1.60 | 26.7^a^ | 25.1^a^ | 1.88 |  | *<0.001* |
| AST, U/L^*,ƚ^ | 17.7^b^ | 23.0^b^ | 4.37 | 87.6^a^ | 103^a^ | 23.2 |  | *<0.001* |
| AST:ALT ratio^*,ƚ,#^ | 1.65^b^ | 1.86^b^ | 0.183 | 3.30^a^ | 3.73^a^ | 0.365 |  | *<0.001* |
| Bilirubin, µmol/L | 1.05^b^ | 1.12^b^ | 0.131 | 5.74^a^ | 5.88^a^ | 0.881 |  | *<0.001* |
| Cholesterol, mmol/L | 0.868 | 0.882 | 0.047 | 0.854 | 0.892 | 0.052 |  | 0.702 |
| Fructose, mmol/L | 2.83^a^ | 3.07^a^ | 0.235 | 1.75^b^ | 1.74^b^ | 0.118 |  | *<0.001* |
| Lactate, mmol/L^ƚ^ | 6.22^a^ | 5.32^a^ | 0.696 | 4.06^b^ | 4.10^b^ | 0.338 |  | *<0.001* |
| NEFA, µmol/L^§,ƚ^ | 37.6^b^ | 38.0^b^ | 4.47 | 154^a^ | 172^a^ | 21.0 |  | *<0.001* |
| Total protein, g/L^*,#^ | 24.4 | 26.1 | 1.52 | 26.1 | 25.8 | 1.41 |  | *<0.001* |
| TG, mmol/L^#^ | 0.085^b^ | 0.113^b^ | 0.017 | 0.367^a^ | 0.385^a^ | 0.062 |  | *<0.001* |
| Urea, mmol/L | 3.77 | 3.88 | 0.238 | 3.55 | 3.71 | 0.150 |  | 0.718 |
| Ig, mg/mL^#^ |  |  |  |  |  |  |  |  |
| IgA |  |  |  | 1.45 | 1.43 | 0.291 |  |  |
| IgG |  |  |  | 3.05 | 3.11 | 0.613 |  |  |
| IgM |  |  |  | 0.653 | 0.734 | 0.156 |  |  |
| IGF-BP, ng/mL |  |  |  |  |  |  |  |  |
| IGF-BP2^#^ |  |  |  | 3006 | 2844 | 321 |  |  |
| IGF-BP3 |  |  |  | 516 | 450 | 54.7 |  |  |
| IGF-BP4 |  |  |  | 165 | 138 | 14.3 |  |  |
| IGF-BP5 |  |  |  | 460 | 476 | 73.3 |  |  |

^1^Values are observed means ± SE; only the largest SE is shown; *n* = 13-17 at 0 h per BiW group, *n* = 19-20 at 4 h per BiW group, except IGF-BP2-5, *n* = 7-9 at 4 h per BiW group.

^2^ALT, Alanine aminotransferase; AST, Aspartate aminotransferase; Ig, Immunoglobulins; IGF-BP, Insulin-like binding protein; NEFA, Non-esterified fatty acids; TG, Triglycerides.

^3^ANOVA *F* Test.

^a,b^Different from time points 0 to 4 h, within BiW group (*P* < 0.05).

^§^Data was log transformed for the 0 h BiW group comparison.

^ƚ^Data was log transformed for the 4 h BiW group comparison.

*Non parametric analysis for the 0 h BiW group comparison, Wilcoxon signed-rank test.

^#^Non parametric analysis for the 4 h BiW group comparison, Wilcoxon signed-rank test.

**Supplementary Table 7:** Plasma free amino acids concentrations of low (LBiW) and normal (NBiW) birthweight (BiW) experimental piglets at birth (0 h) and 4 h post-birth.

| Parameters, µmol/L^1,2^ | 0 h | |  | 4 h | |  | *P*-value^3^ |
| --- | --- | --- | --- | --- | --- | --- | --- |
|  | LBiW | NBiW | SE | LBiW | NBiW | SE | Time |
| Essential-AA^*^ | 892^b^ | 886^b^ | 76.4 | 2041^a^ | 2244^a^ | 213 | *<0.001* |
| Arginine^*^ | 47.4^b^ | 45.8^b^ | 7.03 | 158^a^ | 164^a^ | 17.7 | *<0.001* |
| Phenyalanine^*^ | 54.2^b^ | 49.5^b^ | 7.31 | 160^a^ | 185^a^ | 18.8 | *<0.001* |
| Isoleucine^§^ | 32.6^b^ | 30.4^b^ | 4.75 | 99.3^a^ | 108^a^ | 15.5 | *<0.001* |
| Leucine^§,#^ | 78.3^b^ | 76.8^b^ | 7.65 | 272^a^ | 296^a^ | 34.3 | *<0.001* |
| Lysine | 224^b^ | 257^b^ | 23.9 | 448^a^ | 514^a^ | 46.0 | *<0.001* |
| Valine | 218^b^ | 219^b^ | 15.2 | 417^a^ | 445^a^ | 39.5 | *<0.001* |
| Tryptophan^§^ | 22.0^b^ | 23.1^b^ | 1.32 | 35.8^a^ | 40.1^a^ | 3.45 | *<0.001* |
| Methionine^*,#^ | 8.93^b^ | 6.13^b^ | 1.79 | 77.0^a^ | 76.7^a^ | 14.1 | *<0.001* |
| Non-Essential AA |  |  |  |  |  |  |  |
| Alanine^ƚ,*^ | 833 | 695^b^ | 101 | 828 | 925^a^ | 67.3 | 0.248 |
| Asparagine | 63.1^b^ | 63.9^b^ | 4.49 | 134^a^ | 146^a^ | 12.3 | *<0.001* |
| Aspartate | 12.7^b^ | 12.0^b^ | 1.16 | 54.6^a^ | 41.9^a^ | 7.12 | *<0.001* |
| Cysteine^ƚ^ | 33.5^b^ | 34.0^b^ | 4.80 | 99.4^a^ | 101^a^ | 8.33 | *<0.001* |
| Glutamine | 547^b^ | 569^b^ | 26.5 | 811^a^ | 853^a^ | 42.6 | *<0.001* |
| Glutamate | 127^b^ | 125^b^ | 18.7 | 779^a^ | 606^a^ | 88.5 | *<0.001* |
| Proline | 248^b^ | 226^b^ | 31.8 | 693^a^ | 731^a^ | 71.9 | *<0.001* |
| Total-AA^*^ | 4473^b^ | 4098^b^ | 309 | 7800^a^ | 7923^a^ | 476 | *<0.001* |
| AA Groups |  |  |  |  |  |  |  |
| Arg-Family | 1148^b^ | 1147^b^ | 59.6 | 2860^a^ | 2787^a^ | 186 | *<0.001* |
| ArgbioAv | 102^b^ | 106^b^ | 7.07 | 251^a^ | 226^a^ | 15.8 | *<0.001* |
| Aromatic AA | 159^b^ | 137^b^ | 20.2 | 594^a^ | 656^a^ | 59.9 | *<0.001* |
| Branched-chain AA^*^ | 329^b^ | 326^b^ | 26.9 | 789^a^ | 850^a^ | 87.9 | *<0.001* |
| Gln-Family | 970^b^ | 965^b^ | 54.8 | 2454^a^ | 2366^a^ | 162 | *<0.001* |
| Ketogenic AA | 302^b^ | 334^b^ | 30.1 | 720^a^ | 810^a^ | 77.8 | *<0.001* |
| Non-proteinogenic AA |  |  |  |  |  |  |  |
| Citrulline | 65.7^b^ | 68.1^b^ | 3.02 | 149^a^ | 156^a^ | 10.2 | *<0.001* |
| Ornithine^§^ | 36.6^b^ | 38.0^b^ | 4.49 | 81.7^a^ | 88.7^a^ | 7.51 | *<0.001* |
| Amino-metabolites |  |  |  |  |  |  |  |
| 1-Methylhistidine^§,ƚ^ | 9.13^b^ | 8.36^b^ | 1.03 | 12.4^a^ | 11.3^a^ | 0.833 | 0.004 |
| 3-Methylhistidine | 13.1^b^ | 11.4^b^ | 0.748 | 9.10^a^ | 7.73^a^ | 1.01 | 0.006 |
| AAA | 9.68^b^ | 9.45^b^ | 1.37 | 42.7^a^ | 50.6^a^ | 8.76 | *<0.001* |
| AABA | 28.9 | 29.8 | 1.63 | 30.4 | 32.4 | 1.50 | 0.237 |
| Taurine^ƚ^ | 67.5^b^ | 77.6^b^ | 8.46 | 109^a^ | 124^a^ | 16.1 | 0.030 |

^1^Values are observed means ± SE; only the largest SE is shown; *n* = 13-17 at 0 h per BiW group, *n* = 20 per 4 h BiW group.

^2^AA, Amino acids; AAA, α-aminoadipic acid; AABA, α-aminobutyric acid; Arg-Family, Arginine-family AA; ArgbioAv, Arginine Bioavailability (defined as arginine/(ornithine + citrulline)); Gln-Family, Glutamine-family AA.

^3^ANOVA *F* Test.

^a,b^Different from timepoints 0 to 4 h, within BiW group (*P* < 0.05).

^§^Data was log transformed for the 0 h BiW comparison.

^ƚ^Data was log transformed for the 4 h BiW comparison.

*Non parametric analysis for the 0 h BiW comparison, Wilcoxon signed-rank test.

^#^Non parametric analysis for the 4 h BiW comparison, Wilcoxon signed-rank test.

**Supplementary Table 8:** Zootechnical performance data for pigs with low (LBiW) and normal (NBiW) birthweight (BiW) after weaning (35 to 61 days of age), supplemented with alanine (Ala) or glutamine (Gln) from 1 to 12 days of age.

| Parameters^1,2^ | Age^3^ | LBiW | |  | NBiW | |  | *P*-value^4^ | | |
| --- | --- | --- | --- | --- | --- | --- | --- | --- | --- | --- |
|  |  | Ala | Gln | SE | Ala | Gln | SE | BiW | Day | Interaction |
| BW, kg | 35 | 7.10^h^ | 7.68 | 0.696 | 8.5^g^ | 8.46 | 0.696 | 0.001 | *<0.001* | 0.234 |
|  | 42 | 8.96^f^ | 9.23 | 0.696 | 11.1^e^ | 10.5 | 0.696 |  |  |  |
|  | 49 | 12.1^f^ | 12.2 | 0.700 | 14.2^e^ | 13.4 | 0.700 |  |  |  |
|  | 56 | 13.1^f^ | 13.8^f^ | 0.696 | 16.1^e^ | 15.9^e^ | 0.696 |  |  |  |
|  | 61 | 16.4^f^ | 16.3^f^ | 0.696 | 18.7^e^ | 19.1^e^ | 0.696 |  |  |  |
| ADG, g/d | 35-61 | 341 | 324^h^ | 30.1 | 379 | 376^g^ | 30.1 | 0.039 |  | 0.733 |
| AC, cm | 35 | 46.5 | 46.0 | 1.37 | 48.9 | 48.8 | 1.37 | *<0.001* | *<0.001* | 0.062 |
|  | 42 | 51.7^h^ | 51.1 | 1.37 | 55.0^g^ | 52.9 | 1.37 |  |  |  |
|  | 49 | 58.0^c^ | 54.8^d,f^ | 1.41 | 60.6 | 58.5^e^ | 1.41 |  |  |  |
|  | 56 | 56.3^f^ | 58.0 | 1.37 | 60.5^e^ | 60.7 | 1.37 |  |  |  |
|  | 61 | 57.6 | 54.9^f^ | 1.37 | 59.3 | 60.8^e^ | 1.37 |  |  |  |
| BMI, cm^2^ | 35 | 33.2 | 36.5 | 1.99 | 35.9 | 35.2 | 1.99 | 0.116 | *<0.001* | 0.724 |
|  | 42 | 33.6 | 35.2 | 1.99 | 36.7 | 37.4 | 1.99 |  |  |  |
|  | 49 | 35.6 | 40.0 | 2.10 | 38.9 | 39.1 | 2.10 |  |  |  |
|  | 56 | 40.9 | 40.5 | 1.99 | 43.7 | 43.3 | 1.99 |  |  |  |
|  | 61 | 50.2 | 54.1 | 1.99 | 53.5 | 51.8 | 1.99 |  |  |  |
| CRL, cm | 35 | 47.2 | 47.7 | 1.35 | 49.7 | 48.3 | 1.35 | *<0.001* | *<0.001* | 0.477 |
|  | 42 | 48.3^f^ | 50.3^f^ | 1.35 | 53.0^e^ | 54.4^e^ | 1.35 |  |  |  |
|  | 49 | 53.6^f^ | 54.2^h^ | 1.41 | 58.1^e^ | 57.1^g^ | 1.41 |  |  |  |
|  | 56 | 58.4^f^ | 58.8^h^ | 1.35 | 61.9^e^ | 61.6^g^ | 1.35 |  |  |  |
|  | 61 | 59.3 | 57.5^f^ | 1.35 | 61.4 | 62.8^e^ | 1.35 |  |  |  |
| PI, cm^3^ | 35 | 71.5 | 80.8 | 6.06 | 72.9 | 72.6 | 6.06 | 0.597 | 0.002 | 0.848 |
|  | 42 | 65.1 | 69.5 | 6.06 | 66.6 | 71.2 | 6.06 |  |  |  |
|  | 49 | 61.4 | 74.4 | 6.39 | 64.2 | 67.4 | 6.39 |  |  |  |
|  | 56 | 72.3 | 70.4 | 6.06 | 71.9 | 73.1 | 6.06 |  |  |  |
|  | 61 | 88.6 | 81.9 | 6.06 | 90.7^a^ | 71.0^b^ | 6.06 |  |  |  |
| RT, ^o^C | 35 | 39.2 | 39.0 | 0.187 | 39.2 | 39.3 | 0.187 | 0.170 | 0.001 | 0.557 |
|  | 42 | 39.3 | 39.2 | 0.187 | 39.2 | 39.2 | 0.187 |  |  |  |
|  | 49 | 39.2 | 39.6 | 0.196 | 39.6 | 39.8 | 0.196 |  |  |  |
|  | 56 | 39.5^c^ | 39.0^d^ | 0.187 | 39.3 | 39.2 | 0.187 |  |  |  |
|  | 61 | 39.6 | 39.5 | 0.187 | 39.8 | 39.5 | 0.187 |  |  |  |

^1^Values are LSM ± SE; only the largest SE is shown; *n* = 10 per BiW × Suppl group.

^2^BW, bodyweight; ADG, average daily gain; AC, abdominal circumference; BMI, bodymass index; CRL, crown-rump length; PI, ponderal index; RT, rectal temperature; Suppl, Supplementation.

^3^Age, piglet age in days.

^4^ANOVA *F* Test.

^a,b^Different from Ala-supplemented pigs within BiW group (*P*< 0.05).

^c,d^Tend to be different from Ala-supplemented pigs within BiW group (*P* < 0.10).

^e,f^Different from NBiW pigs within Suppl group (*P* < 0.05).

^g,h^Tend to be different from NBiW pigs within Suppl group (*P* < 0.10).

**Supplementary Table 9:** Pre- (27 days of age) to early post-weaning (32 days of age) concentrations of reduced (GSH) and oxidized (GSSG) glutathione in red blood cells of low (LBiW) and normal (NBiW) birthweight (BiW) pigs supplemented with alanine (Ala) or glutamine (Gln) from 1 to 12 days of age.

| Parameters^1^ | Age^2^ | LBiW | |  | NBiW | |  | *P*-value^3^ |
| --- | --- | --- | --- | --- | --- | --- | --- | --- |
|  |  | Ala | Gln |  | Ala | Gln | SE | Day |
| GSH, mM | 27 | 1.75^i^ | 1.64^i^ |  | 1.71^i^ | 1.71^i^ | 0.091 | *< 0.001* |
|  | 29 | 1.53 | 1.53 |  | 1.58 | 1.50 | 0.096 |  |
|  | 30 | 1.34 | 1.40 |  | 1.45 | 1.40 | 0.091 |  |
|  | 32 | 1.23^b,j^ | 1.35^j^ |  | 1.42^a,j^ | 1.40^j^ | 0.091 |  |
| GSSG, µM | 27 | 20.0^b^ | 23.4 |  | 40.2^a,e^ | 19.7^f^ | 8.89 | 0.012 |
|  | 29 | 16.5 | 21.1 |  | 10.9 | 16.7 | 10.0 |  |
|  | 30 | 46.8^a,g^ | 29.7^h^ |  | 25.3^b^ | 24.4 | 9.31 |  |
|  | 32 | 27.4 | 14.0 |  | 31.1 | 17.2 | 8.84 |  |
| GSH:GSSG ratio, mM/mM | 27 | 159 | 159 |  | 142 | 166 | 33.4 | 0.003 |
|  | 29 | 154 | 157 |  | 196 | 162 | 33.6 |  |
|  | 30 | 107 | 135 |  | 109 | 128 | 33.3 |  |
|  | 32 | 111 | 154 |  | 134 | 148 | 32.5 |  |
| Total GSH, mM | 27 | 1.80^i^ | 1.68^i^ |  | 1.79^i^ | 1.74^i^ | 0.089 | *< 0.001* |
|  | 29 | 1.58 | 1.57 |  | 1.65 | 1.51 | 0.093 |  |
|  | 30 | 1.43 | 1.45 |  | 1.53 | 1.46 | 0.088 |  |
|  | 32 | 1.30^d,j^ | 1.38^j^ |  | 1.48^c,j^ | 1.43^j^ | 0.088 |  |

^1^Values are LSM ± SE; only the largest SE is shown; GSH, GSH:GSSG ratio, Total GSH *n* = 7-9; GSSG *n* = 5-9 per BiW x Suppl group.

^2^Age, piglet age in days, with weaning at 28 days of age.

^3^ANOVA *F* Test.

^a,b^Different from Ala-supplemented pigs within BiW group (*P* < 0.05).

^c,d^Tend to be different from Ala-supplemented pigs within BiW group (*P* < 0.10).

^e,f^Different from NBiW pigs within supplementation group (*P* < 0.05).

^g,h^Tend to be different from NBiW pigs within supplementation group (*P*< 0.10).

^i,j^Different from prior to weaning (27 d) to post-weaning (32 d) (*P* < 0.05).

**Supplementary Table 10:** Plasma concentrations of free amino acids and amino-metabolites within experimental group altered from the pre- (27 d) to post-weaning (29, 30 and 32 d) using one-way ANOVA analysis and Tukey’s HSD test.

| Experimental group | Parameters^1^ | *P*-value | Post-hoc (Tukey’s HSD) |
| --- | --- | --- | --- |
| LBiW-Gln | α-aminoadipic acid | 2.96E-04 | 30 d-27 d; 32 d-27 d; 30 d-29 d; 32 d-29 d |
|  | ArgbioAv | 2.71E-03 | 30 d-27 d; 32 d-27 d |
|  | Arginine | 7.51E-03 | 32 d-27 d |
|  | Aromatic AA | 8.52E-04 | 30 d-27 d; 32 d-27 d |
|  | Branched chain AA | 1.24E-04 | 30 d-27 d; 32 d-27 d; 30 d-29 d; 32 d-29 d |
|  | Citrulline | 3.19E-03 | 30 d-27 d; 32 d-27 d |
|  | Essential AA | 2.40E-02 |  |
|  | Glutamine | 1.20E-04 | 29 d-27 d; 32 d-29 d |
|  | Histidine | 6.93E-04 | 29 d-27 d; 30 d-27 d; 32 d-27 d |
|  | Ketogenic AA | 6.22E-05 | 30 d-27 d; 32 d-27 d; 30 d-29 d; 32 d-29 d |
|  | Leucine | 1.27E-05 | 30 d-27 d; 32 d-27 d; 30 d-29 d; 32 d-29 d |
|  | Lysine | 1.43E-03 | 30 d-27 d; 32 d-27 d |
|  | Phenylalanine | 1.32E-02 | 32 d-29 d |
|  | Proline | 5.72E-07 | 29 d-27 d; 30 d-27 d; 32 d-27 d |
|  | Serine | 1.38E-04 | 29 d-27 d; 30 d-27 d; 32 d-27 d |
|  | Tryptophan | 1.14E-03 | 29 d-27 d; 30 d-27 d; 32 d-27 d |
|  | Tyrosine | 8.49E-04 | 29 d-27 d; 30 d-27 d; 32 d-27 d |
|  | Valine | 3.86E-06 | 32 d-27 d; 30 d-29 d; 32 d-29 d |
| LBiW-Ala | α-aminobutyric acid | 4.59E-03 | 29 d-27 d |
|  | ArgbioAv | 7.53E-03 | 30 d-27 d; 32 d-27 d |
|  | Aromatic-AA | 9.85E-04 | 30 d-27 d; 32 d-27 d |
|  | Branched chain-AA | 2.08E-02 | 32 d-27 d; 32 d-29 d |
|  | Citrulline | 2.72E-02 | 32 d-27 d |
|  | Glutamine | 1.01E-03 | 29 d-27 d; 32 d-29 d |
|  | Histidine | 8.99E-05 | 29 d-27 d; 30 d-27 d; 32 d-27 d |
|  | Ketogenic-AA | 1.42E-03 | 30 d-27 d; 32 d-27 d; 32 d-29 d |
|  | Leucine | 2.48E-02 | 32 d-29 d |
|  | Lysine | 6.86E-04 | 30 d-27 d; 32 d-27 d |
|  | Ornithine | 1.12E-02 | 30 d-27 d; 32 d-27 d |
|  | Proline | 1.79E-08 | 29 d-27 d; 30 d-27 d; 32 d-27 d |
|  | Serine | 2.33E-04 | 29 d-27 d; 30 d-27 d; 32 d-27 d |
|  | Threonine | 1.56E-03 | 30 d-27 d; 32 d-27 d; 32 d-29 d |
|  | Total-AA | 3.13E-02 | 29 d-27 d |
|  | Tryptophan | 6.56E-04 | 30 d-27 d; 32 d-27 d |
|  | Tyrosine | 8.04E-04 | 29 d-27 d; 30 d-27 d; 32 d-27 d |
| NBiW-Gln | Arginine | 1.31E-02 | 32 d-27 d; 32 d-29 d |
|  | Aromatic-AA | 1.43E-02 | 32 d-27 d |
|  | Branched chain-AA | 1.77E-02 | 32 d-29 d |
|  | Citrulline | 1.71E-03 | 29 d-27 d; 30 d-27 d; 32 d-27 d |
|  | Leucine | 7.08E-04 | 30 d-27 d; 32 d-27 d; 30 d-29 d; 32 d-29 d |
|  | Threonine | 7.47E-03 | 30 d-27 d; 32 d-27 d |
|  | Tryptophan | 1.05E-02 | 32 d-27 d |
|  | Tyrosine | 6.67E-03 | 29 d-27 d; 30 d-27 d; 32 d-27 d |
|  | Valine | 7.29E-05 | 32 d-27 d; 30 d-29 d; 32 d-29 d |
| NBiW-Ala | ArgbioAv | 4.02E-03 | 29 d-27 d; 30 d-27 d; 32 d-27 d |
|  | Aromatic-AA | 1.09E-03 | 30 d-27 d; 32 d-27 d |
|  | Citrulline | 9.92E-04 | 29 d-27 d; 30 d-27 d; 32 d-27 d |
|  | Glutamine | 5.90E-03 | 32 d-29 d |
|  | Histidine | 1.38E-02 | 30 d-27 d; 32 d-27 d |
|  | Ketogenic-AA | 3.84E-03 | 30 d-27 d; 32 d-27 d |
|  | Lysine | 4.22E-04 | 29 d-27 d; 30 d-27 d; 32 d-27 d |
|  | Proline | 1.88E-07 | 29 d-27 d; 30 d-27 d; 32 d-27 d |
|  | Taurine | 3.19E-04 | 29 d-27 d; 30 d-27 d; 32 d-27 d |
|  | Threonine | 1.38E-03 | 30 d-27 d; 32 d-27 d |
|  | Tryptophan | 2.13E-05 | 29 d-27 d; 30 d-27 d; 32 d-27 d |
|  | Tyrosine | 3.38E-04 | 30 d-27 d; 32 d-27 d |

^1^AA, Amino acid; Ala, Alanine; ArgbioAv, Arginine Bioavailability (defined as arginine/(ornithine + citrulline)); Gln, Glutamine; LBiW, Low birthweight; NBiW, Normal birthweight.

**Supplementary Table 11:** Bodyweight and rectal temperature of low (LBiW) and normal (NBiW) birthweight (BiW) pigs supplemented with alanine (Ala) or glutamine (Gln) from 1 to 12 days of age, following an intraperitoneal injection of LPS (100 µg/kg bodyweight) at 55 days of age.

| Parameters^1,2^ | Time^3^ | LBiW | |  | NBiW | |  | *P*-value^4^ | | |
| --- | --- | --- | --- | --- | --- | --- | --- | --- | --- | --- |
|  |  | Ala | Gln | SE | Ala | Gln | SE | BiW | Suppl | Time |
| BW, kg | -15 | 14.7^d,g^ | 14.5^d,g^ | 0.902 | 17.0^c,g^ | 16.4^c,g^ | 0.651 | 0.005 | 0.894 | *< 0.001* |
|  | 60 | 14.3^d^ | 14.3^f^ | 0.860 | 16.6^c^ | 15.8^e^ | 0.627 |  |  |  |
|  | 180 | 13.9^d^ | 14.0^f^ | 0.861 | 16.2^c^ | 15.6^e^ | 0.705 |  |  |  |
|  | 360 | 14.1^d^ | 13.8^d^ | 0.883 | 16.2^c^ | 15.6^c^ | 0.728 |  |  |  |
|  | 720 | 13.7^d^ | 13.8^f^ | 0.827 | 15.9^c^ | 15.5^e^ | 0.697 |  |  |  |
|  | 1440 | 13.3^d,h^ | 13.5^f,h^ | 0.823 | 15.6^c,h^ | 15.2^e,h^ | 0.738 |  |  |  |
| RT, ^o^C | -15 | 39.4 | 39.6 | 0.116 | 39.3 | 39.4 | 0.101 | 0.670 | 0.041 | 0.024 |
|  | 60 | 39.9 | 40.1 | 0.231 | 39.7 | 40.0 | 0.350 |  |  |  |
|  | 180 | 39.7 | 40.2 | 0.164 | 39.6 | 39.5 | 0.381 |  |  |  |
|  | 360 | 38.8^b^ | 40.3^a^ | 0.418 | 39.4 | 39.7 | 0.522 |  |  |  |
|  | 720 | 39.1 | 39.6 | 0.405 | 39.6 | 39.6 | 0.298 |  |  |  |
|  | 1440 | 39.5 | 39.8 | 0.321 | 39.6 | 39.9 | 0.263 |  |  |  |

^1^Values are observed means ± SE; only the largest SE is shown; *n* = 10 per BiW x Suppl group.

^2^BW, Bodyweight; RT, Rectal temperature; Suppl, Supplementation.

^3^Time, min relative to LPS injection.

^4^ANOVA *F* Test.

^a,b^Different from Ala-supplemented pigs within BiW group (*P* < 0.05).

^c,d^Different from NBiW pigs within Suppl group (*P*< 0.05).

^e,f^Tend to be different from NBiW pigs within Suppl group (*P* < 0.10).

^g,h^Different from prior (0) to 1440 min post-LPS injection (*P*< 0.05).

**Supplementary Table 12:** Concentrations of reduced (GSH) and oxidized (GSSG) glutathione in red blood cells of low (LBiW) and normal (NBiW) birthweight (BiW) pigs supplemented with alanine (Ala) or glutamine (Gln) from 1 to 12 days of age following an intraperitoneal injection of LPS (100 µg/kg bodyweight) at 55 days of age.

| Parameters^1^ | Time^2^ | LBiW | |  | NBiW | |  | *P*-value^3^ | |
| --- | --- | --- | --- | --- | --- | --- | --- | --- | --- |
|  |  | Ala | Gln |  | Ala | Gln | SE | Time | Interaction |
| GSH, mM | -15 | 1.91^f^ | 1.94 |  | 1.98^f^ | 1.98 | 0.131 | 0.012 | 0.704 |
|  | 60 | 2.03 | 2.12 |  | 2.10 | 2.06 | 0.129 |  |  |
|  | 360 | 2.04 | 2.05 |  | 2.13^e^ | 2.00 | 0.139 |  |  |
|  | 720 | 2.13^e^ | 1.92 |  | 2.13^a^ | 1.86^b^ | 0.167 |  |  |
| GSSG, µM | -15 | 38.2 | 28.7 |  | 33.1^f^ | 32.3 | 12.1 | 0.002 | 0.516 |
|  | 60 | 49.3 | 40.9 |  | 38.7 | 39.2 | 17.8 |  |  |
|  | 360 | 51.8 | 39.0 |  | 52.5^a,e^ | 30.4^b^ | 14.2 |  |  |
|  | 720 | 38.2^c^ | 33.4 |  | 18.1^d^ | 23.3 | 10.9 |  |  |
| GSH:GSSG Ratio, mM/mM | -15 | 172 | 220 |  | 76.2^f^ | 155 | 67.9 | 0.535 | 0.069 |
|  | 60 | 166 | 132 |  | 116 | 260 | 107 |  |  |
|  | 360 | 174 | 196 |  | 111 | 238 | 94.6 |  |  |
|  | 720 | 77.6^d^ | 176 |  | 293^c,e^ | 212 | 76.5 |  |  |
| Total GSH, mM | -15 | 1.93 | 2.05 |  | 2.17^f^ | 2.05 | 0.123 | 0.042 | 0.463 |
|  | 60 | 2.13 | 1.94 |  | 2.19 | 2.13 | 0.189 |  |  |
|  | 360 | 2.09 | 2.13 |  | 2.23^e^ | 2.07 | 0.127 |  |  |
|  | 720 | 2.20 | 1.98 |  | 2.24 | 1.91 | 0.171 |  |  |

^1^Values are observed means ± SE; only the largest SE is shown. GSH *n* =5-9; GSH:GSSG ratio, Total GSH, GSSG *n* = 6-9 per BiW x Suppl group.

^2^Time, min relative to LPS injection.

^3^ANOVA *F* Test. Interaction is BiW × Supplementation (Suppl) × Day.

^a,b^Tend to be different from Ala-supplemented pigs within BiW group (*P* < 0.10).

^c,d^Tend be different from NBiW pigs within Suppl group (*P* < 0.10).

^e,f^Tend to be different prior (-15) to the LPS injection (*P* < 0.10).

**Supplementary Table 13:** Plasma concentrations of free amino acids and amino-metabolites within experimental group following an intraperitoneal injection of LPS (100 µg/kg BW) at 55 days of age using one-way ANOVA analysis and Tukey’s HSD test.

| Experimental group | Parameters^1^ |  | *P*-value | Post-hoc (Tukey’s HSD) |
| --- | --- | --- | --- | --- |
| LBiW-Gln | 3-Methylhistidine |  | 4.98E-03 | 12 h-Basal; 12 h-1 h; 12 h-3 h |
|  | α-aminoadipic acid |  | 7.50E-06 | 12 h-Basal; 12 h-1 h; 12 h-3 h |
|  | Arginine |  | 2.54E-04 | 12 h-Basal; 12 h-1 h; 6 h-3 h; 12 h-3 h |
|  | Citrulline |  | 2.40E-03 | 12 h-Basal; 12 h-1 h |
|  | Ketogenic-AA |  | 5.44E-06 | 12 h-Basal; 6 h-1 h; 12 h-1 h; 6 h-3 h; 12 h-3 h |
|  | Leucine |  | 2.66E-08 | 6 h-Basal; 12 h-Basal; 6 h-1 h; 12 h-1 h; 6 h-3 h; 12 h-3 h |
|  | Phenylalanine |  | 8.66E-15 | 1 h-Basal; 3 h-Basal; 12 h-Basal; 6 h-1 h; 12 h-1 h; 6 h-3 h; 12 h-3 h; 12 h-6 h |
|  | Tryptophan |  | 9.64E-06 | 3 h-Basal; 6 h-Basal; 12 h-Basal; 6 h-1 h; 12 h-1 h |
|  | Tyrosine |  | 3.62E-03 | 3 h-Basal |
|  | Valine |  | 3.82E-06 | 12 h-Basal; 12 h-1 h; 6 h-3 h; 12 h-3 h |
| LBiW-Ala | α-aminoadipic acid |  | 6.84E-08 | 12 h-1 h; 6 h-1 h; 3 h-12 h; 6 h-12 h; Basal-12 h; Basal-6 h |
|  | α-aminobutyric acid |  | 8.29E-06 | 12 h-1 h; 3 h-12 h; 6 h-12 h; Basal-12 h |
|  | Alanine |  | 1.82E-05 | 6 h-1 h; Basal-12 h; Basal-3 h; Basal-6 h |
|  | ArgbioAv |  | 5.88E-05 | 12 h-1 h; 6 h-1 h; 3 h-12 h; Basal-12 h; 6 h-3 h |
|  | Arginine-Family |  | 1.20E-02 | 3 h-12 h |
|  | Citrulline |  | 3.79E-05 | 12 h-1 h; 6 h-1 h; 3 h-12 h; Basal-12 h; 6 h-3 h; Basal-6 h |
|  | γ-aminobutyric acid |  | 3.10E-05 | 12 h-1 h; 6 h-1 h; Basal-12 h; Basal-6 h |
|  | Glucogenic-AA |  | 1.52E-04 | 6 h-1 h; Basal-12 h; 6 h-3 h; Basal-6 h |
|  | Histidine |  | 7.47E-04 | 12 h-1 h; 3 h-12 h; Basal-12 h |
|  | Isoleucine |  | 2.36E-02 | Basal-12 h |
|  | Ketogenic-AA |  | 2.59E-06 | 12 h-1 h; 6 h-1 h; 3 h-12 h; Basal-12 h; 6 h-3 h |
|  | Leucine |  | 2.40E-06 | 12 h-1 h; 6 h-1 h; 3 h-12 h; Basal-12 h; 6 h-3 h |
|  | Phenylalanine |  | 9.00E-11 | 12 h-1 h; 6 h-1 h; 3 h-12 h; 6 h-12 h; Basal-12 h; 6 h-3 h; Basal-3 h |
|  | Serine |  | 3.48E-06 | 12 h-1 h; 6 h-1 h; 3 h-12 h; Basal-12 h; 6 h-3 h; Basal-6 h |
|  | Taurine |  | 2.27E-03 | 12 h-1 h; Basal-12 h; Basal-6 h |
|  | Total-AA |  | 1.27E-04 | 12 h-1 h; 6 h-1 h; 3 h-12 h; Basal-12 h; 6 h-3 h; Basal-6 h |
|  | Tryptophan |  | 6.06E-10 | 12 h-1 h; 3 h-1 h; 6 h-1 h; Basal-12 h; 6 h-3 h; Basal-3 h; Basal-6 h |
|  | Tyrosine |  | 3.62E-04 | 3 h-12 h; Basal-3 h; Basal-6 h |
|  | β-Alanine |  | 7.58E-04 | 12 h-1 h; 3 h-12 h; Basal-12 h |
| NBiW-Gln | α-aminoadipic acid |  | 6.21E-05 | 12 h-Basal; 12 h-1 h; 12 h-3 h |
|  | Arginine |  | 2.29E-04 | 12 h-Basal; 6 h-1 h; 12 h-1 h; 12 h-3 h |
|  | β-Alanine |  | 7.78E-04 | 6 h-Basal; 12 h-Basal; 12 h-1 h; 12 h-3 h |
|  | Citrulline |  | 1.31E-03 | 6 h-Basal; 12 h-Basal; 6 h-1 h; 12 h-1 h |
|  | γ-aminobutyric acid |  | 2.51E-04 | 3 h-Basal; 6 h-Basal; 12 h-Basal; 6 h-1 h |
|  | Histidine |  | 4.97E-05 | 6 h-Basal; 12 h-Basal; 12 h-1 h; 12 h-3 h |
|  | Isoleucine |  | 4.02E-03 | 3 h-Basal; 6 h-Basal; 12 h-Basal |
|  | Tryptophan |  | 4.27E-03 | 6 h-Basal; 6 h-1 h |
|  | Tyrosine |  | 4.87E-03 | 3 h-Basal; 12 h-3 h |
|  | Valine |  | 4.97E-05 | 12 h-Basal; 6 h-1 h; 12 h-1 h; 6 h-3 h; 12 h-3 h |
| NBiW-Ala | α-aminoadipic acid |  | 9.45E-07 | 12 h-Basal; 12 h-1 h; 12 h-3 h; 12 h-6 h |
|  | Alanine |  | 2.30E-04 | 6 h-Basal; 12 h-Basal; 6 h-1 h |
|  | Aspartate |  | 7.10E-03 | 3 h-Basal; 6 h-Basal |
|  | γ-aminobutyric acid |  | 4.67E-06 | 6 h-Basal; 12 h-Basal; 6 h-1 h; 12 h-1 h |
|  | Isoleucine |  | 1.29E-03 | 3 h-Basal; 6 h-Basal; 12 h-Basal |
|  | Ketogenic-AA |  | 6.86E-06 | 12 h-Basal; 6 h-1 h; 12 h-1 h; 6 h-3 h; 12 h-3 h |
|  | Serine |  | 9.14E-05 | 6 h-Basal; 12 h-Basal; 6 h-1 h; 12 h-1 h |
|  | Taurine |  | 3.56E-03 | 12 h-Basal; 12 h-1 h |
|  | Tryptophan |  | 1.83E-11 | 3 h-Basal; 6 h-Basal; 12 h-Basal; 3 h-1 h; 6 h-1 h; 12 h-1 h; 6 h-3 h; 12 h-3 h |
|  | Tyrosine |  | 4.59E-04 | 3 h-Basal; 6 h-Basal; 12 h-3 h |
|  | β-Alanine |  | 1.01E-04 | 12 h-Basal; 12 h-1 h; 12 h-3 h |

^1^AA, Amino acid; Ala, Alanine; ArgbioAv, Arginine Bioavailability (defined as arginine/(ornithine + citrulline)); Gln, Glutamine; LBiW, Low birthweight; NBiW, Normal birthweight.
